# Supplementary figures and images for: The GNU subunit of PNG kinase, the developmental regulator of mRNA translation, binds BIC-C to localize to RNP granules
Source: eLife. 2021 Jul 12;10:e67294. doi: 10.7554/eLife.67294 (PMC8313231; doi:10.7554/eLife.67294)

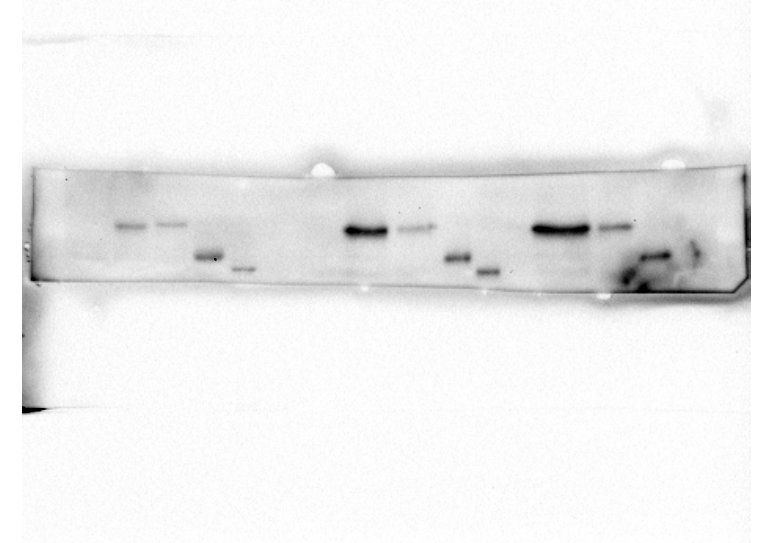

Supplement: Figure 1—source data 1. [file elife-67294-fig1-data1.zip › Figure 1A_Source data 1/Figure 1A-source data 1 raw anti-GFP immunoblot.tif]

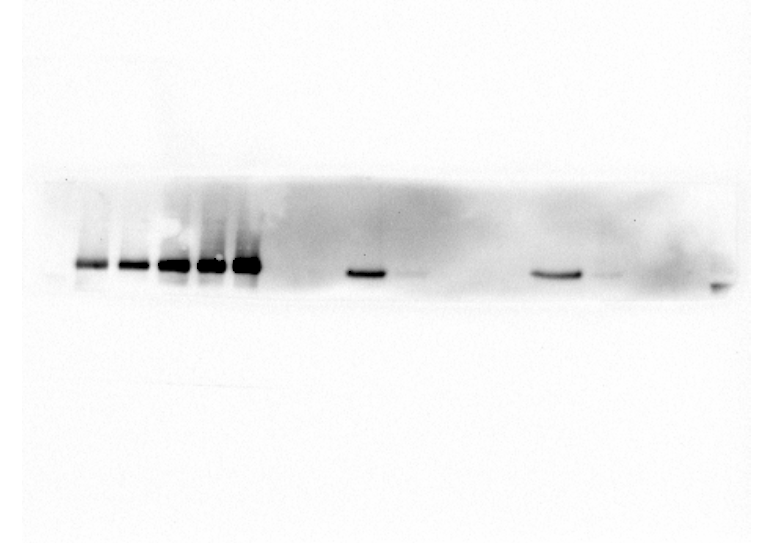

Supplement: Figure 1—source data 1. [file elife-67294-fig1-data1.zip › Figure 1A_Source data 1/Figure 1A-source data 1 raw anti-BIC-C immunoblot.tif]

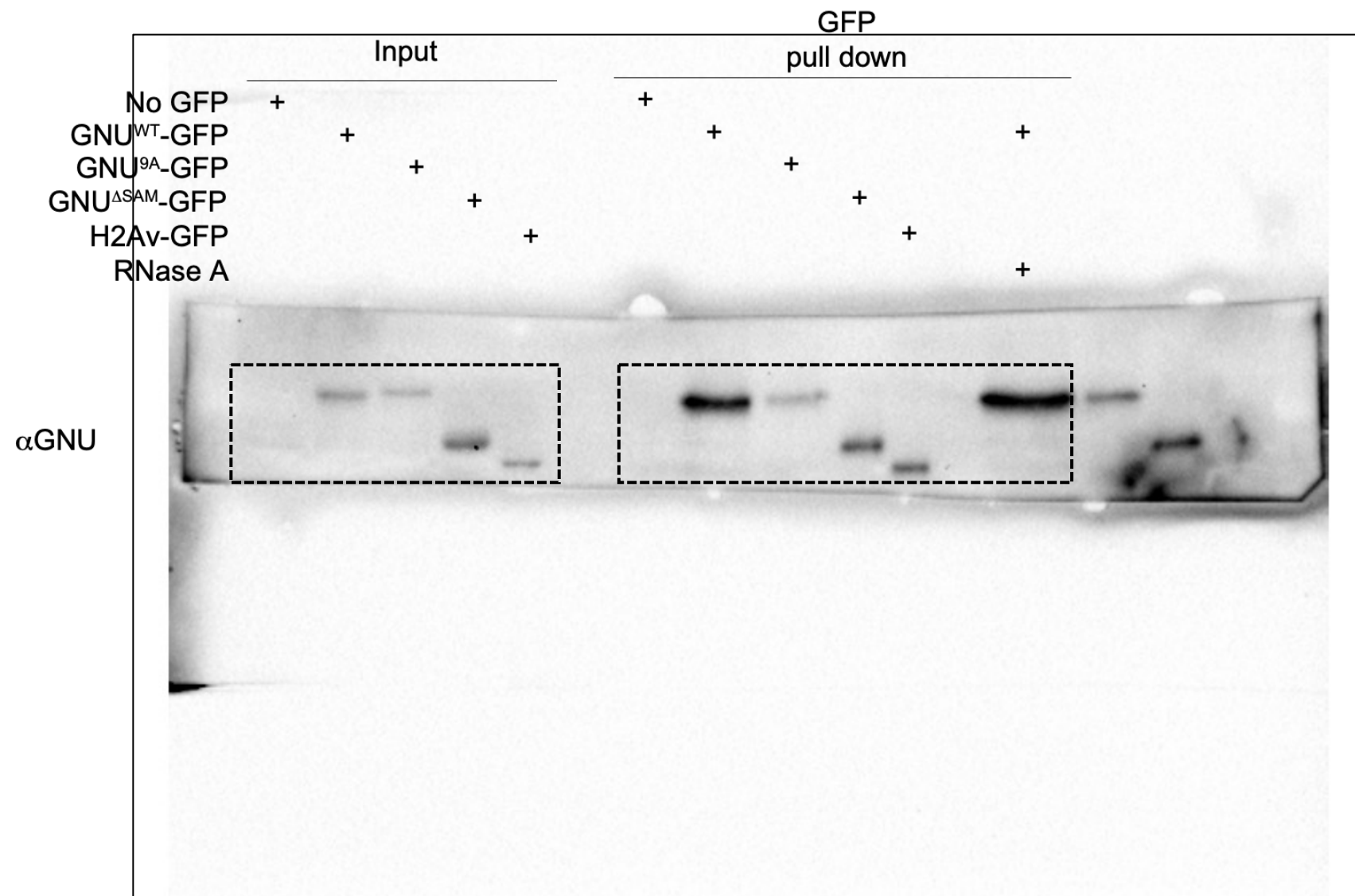

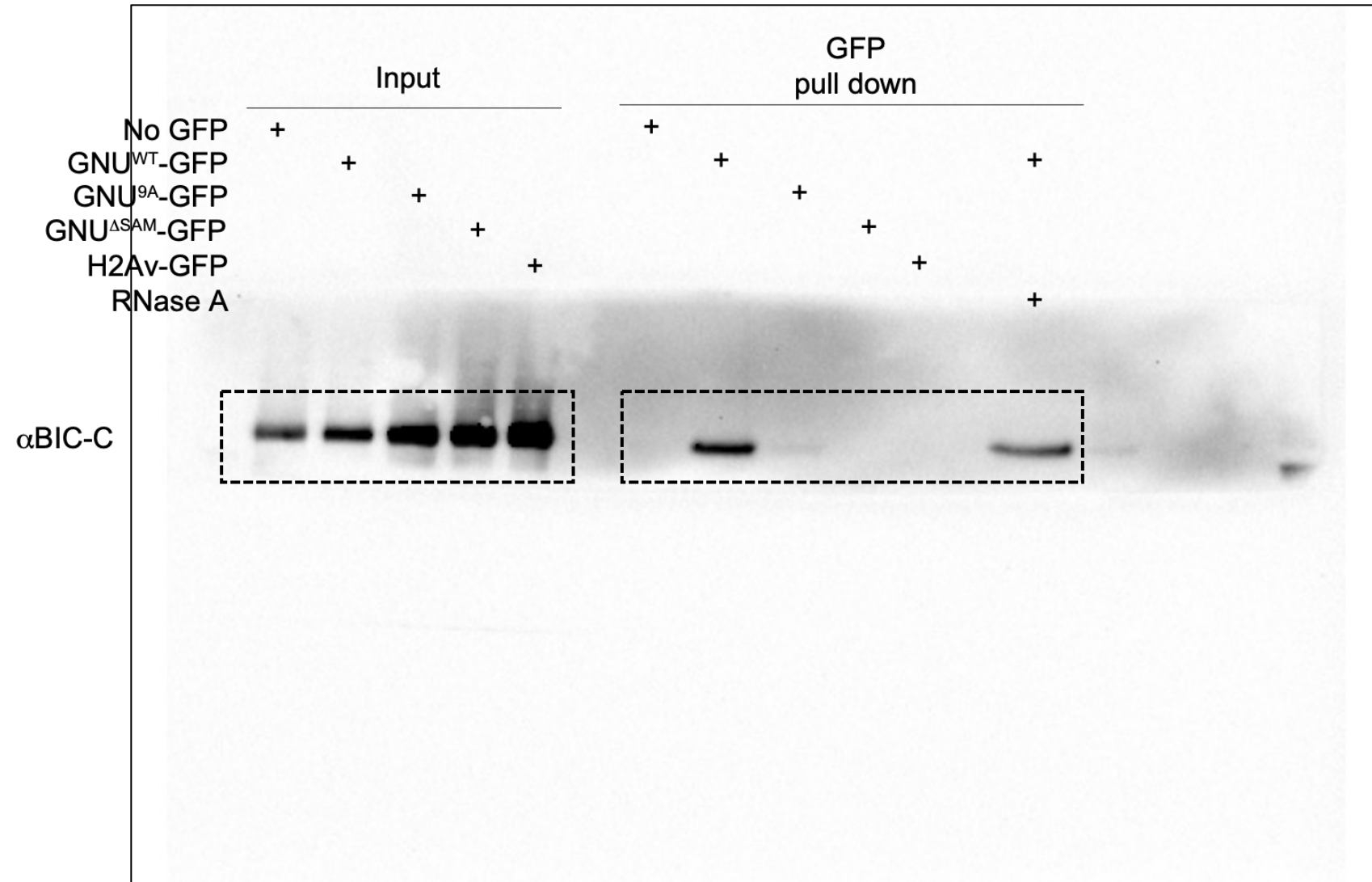

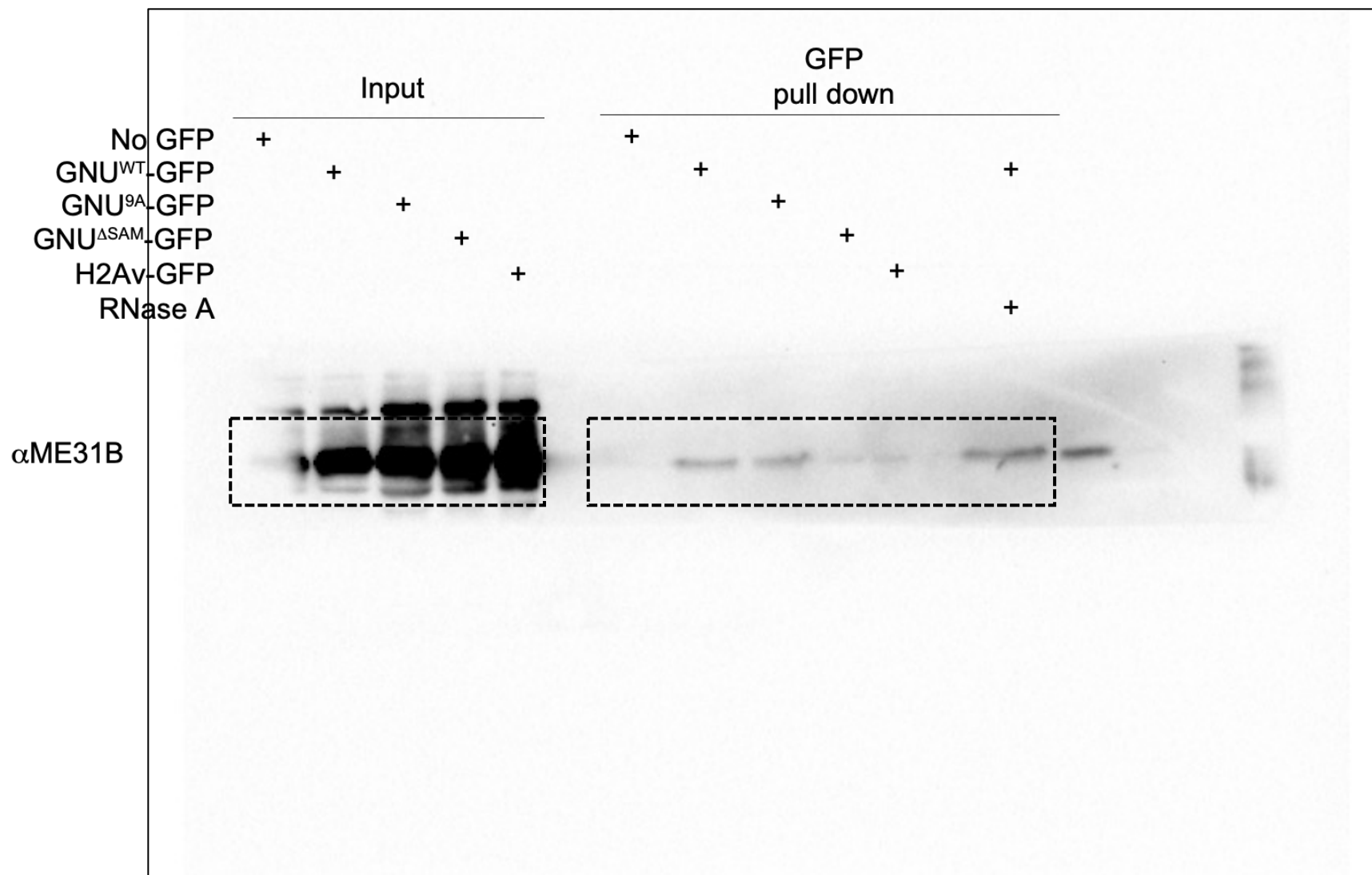

Supplement: Figure 1—source data 1. [file elife-67294-fig1-data1.zip › Figure 1A_Source data 1/Figure 1A_Source data 1 labeled bands.pdf]

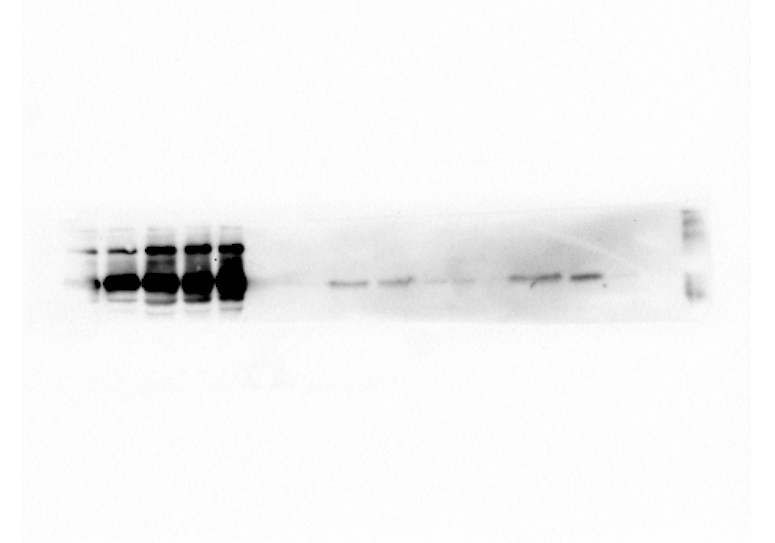

Supplement: Figure 1—source data 1. [file elife-67294-fig1-data1.zip › Figure 1A_Source data 1/Figure 1A-source data 1 raw anti-ME31B immunoblot.tif]

Figure 2A-Source data

$\alpha$ MBP

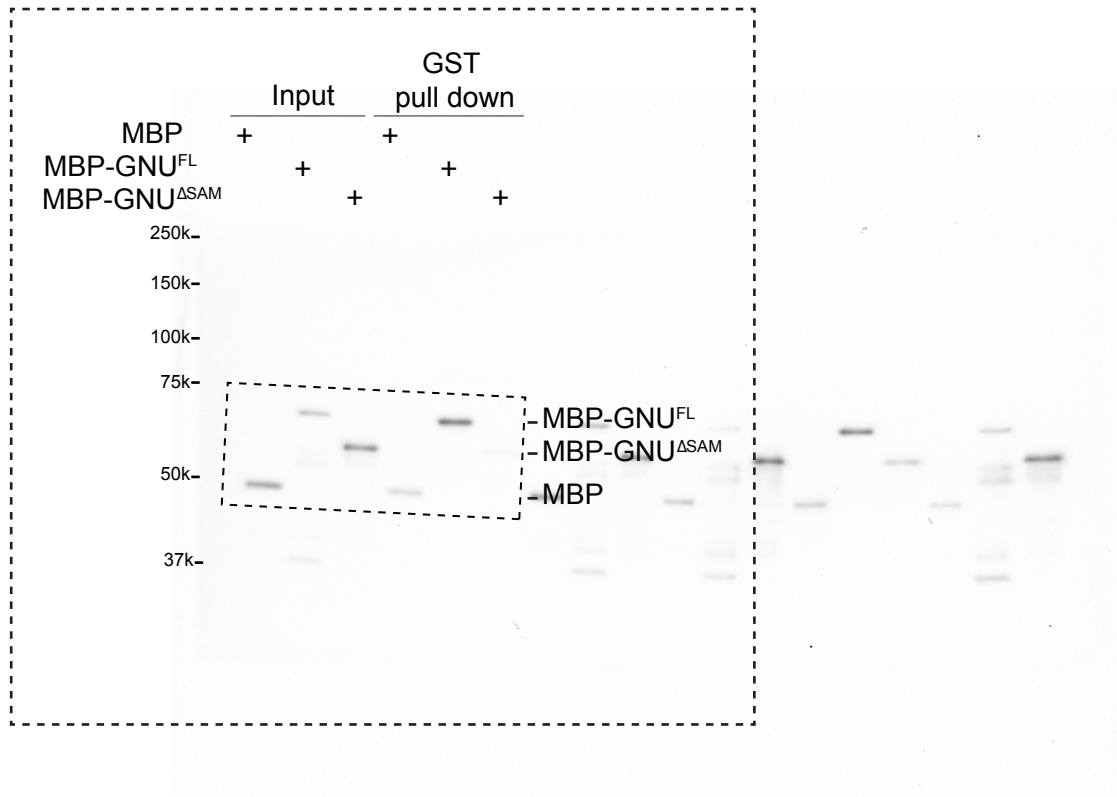

$\alpha$ GST

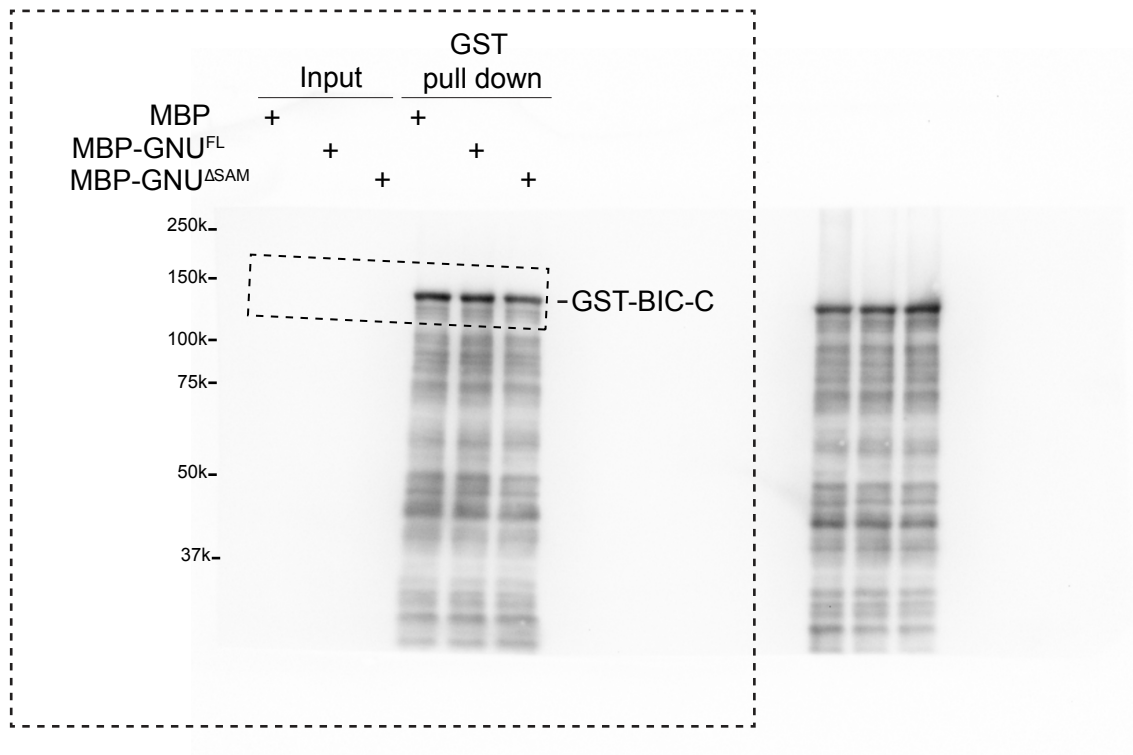

Supplement: Figure 2—source data 1. [file elife-67294-fig2-data1.zip › Fig2A_source_data/Figure 2A-Source-data 1 labeled bands.pdf]

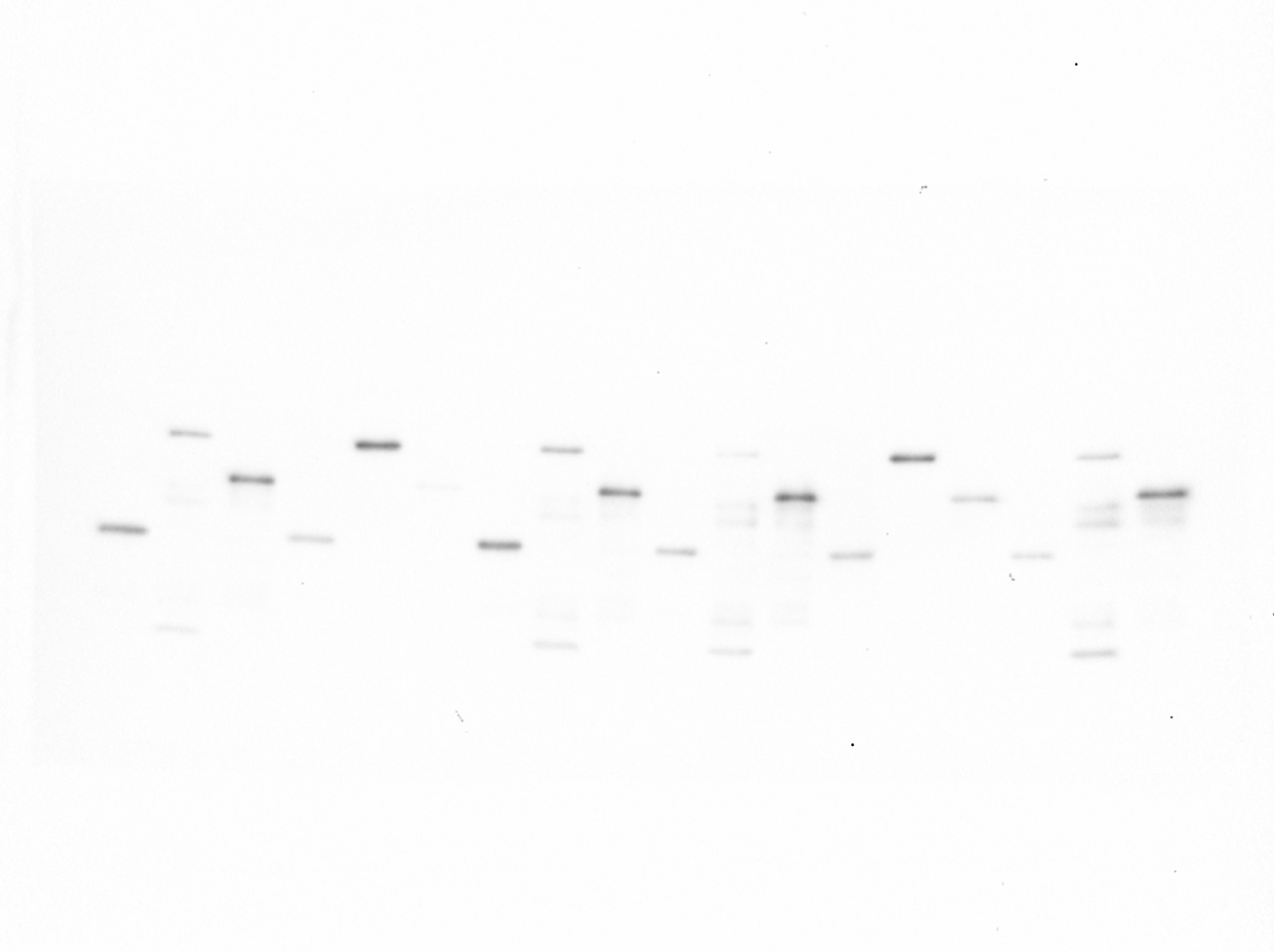

Supplement: Figure 2—source data 1. [file elife-67294-fig2-data1.zip › Fig2A_source_data/Figure 2A-source data 1 raw MBP immunoblot_small.tif]

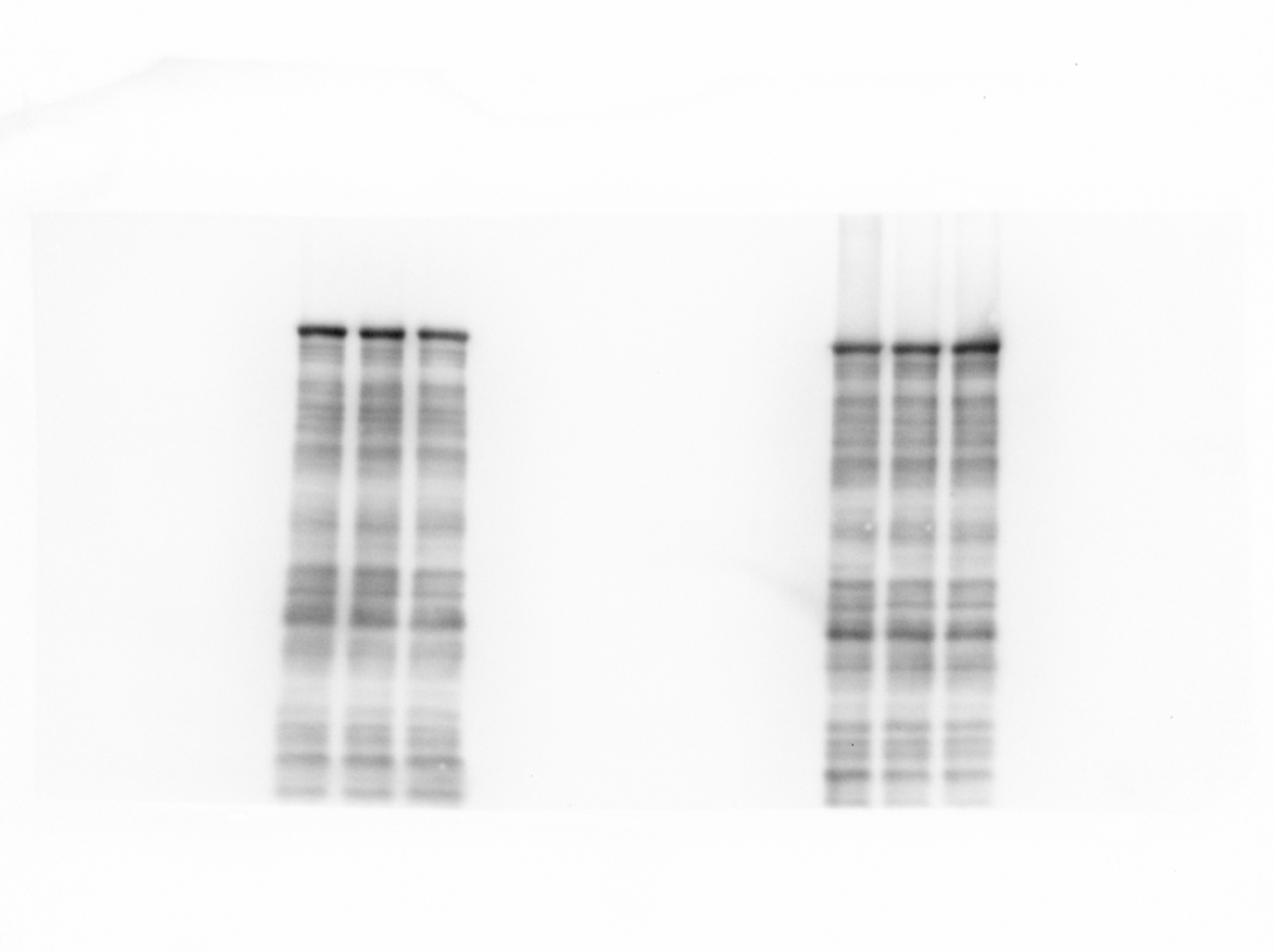

Supplement: Figure 2—source data 1. [file elife-67294-fig2-data1.zip › Fig2A_source_data/Figure 2A-source data 1 raw GST immunoblot_small.tif]

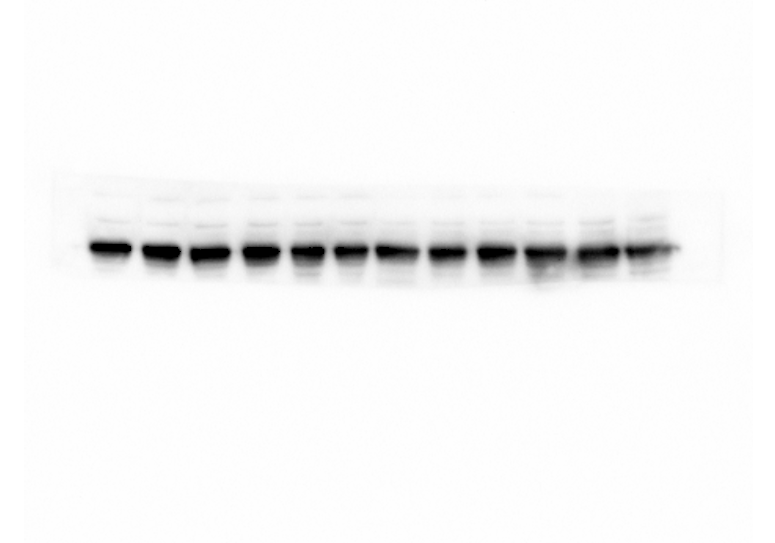

Supplement: Figure 2—source data 3. [file elife-67294-fig2-data3.zip › Figure 2D_Source data 1/Figure 2D-Source data 1 raw anti-ME31B immunoblot.tif]

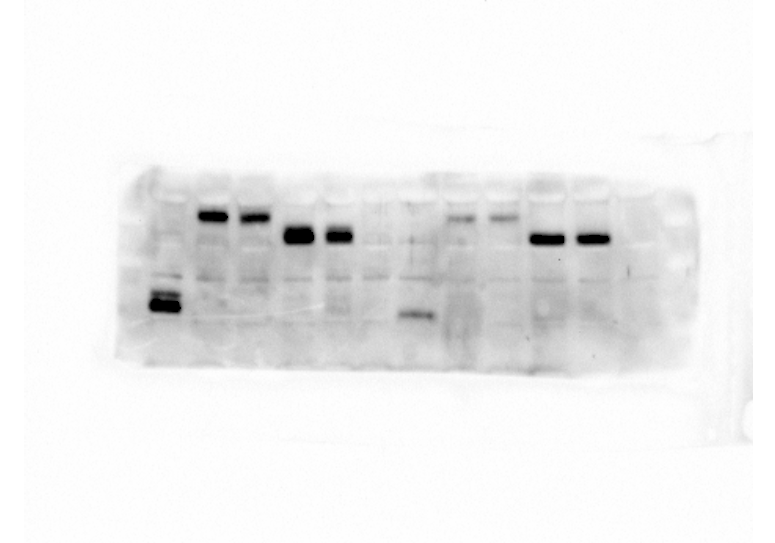

Supplement: Figure 2—source data 3. [file elife-67294-fig2-data3.zip › Figure 2D_Source data 1/Figure 2D-Source data 1 raw anti-GNU immunoblot.tif]

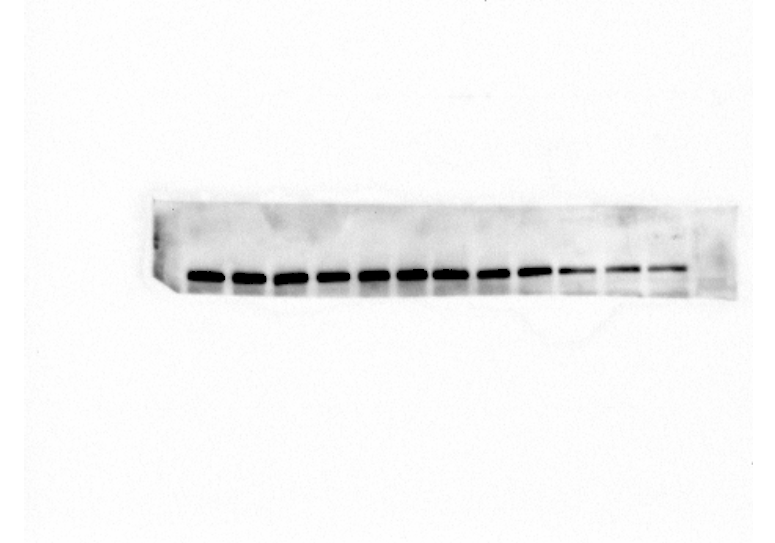

Supplement: Figure 2—source data 3. [file elife-67294-fig2-data3.zip › Figure 2D_Source data 1/Figure 2D-Source data 1 raw anti-BIC-C immunoblot.tif]

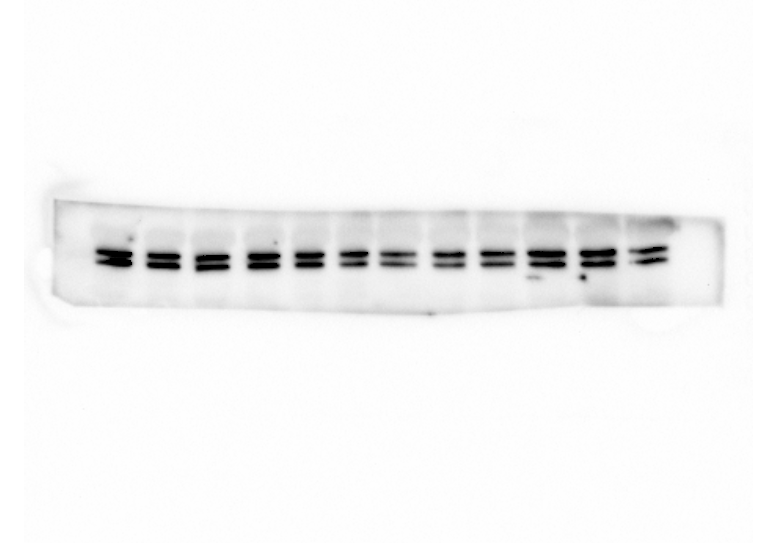

Supplement: Figure 2—source data 3. [file elife-67294-fig2-data3.zip › Figure 2D_Source data 1/Figure 2D-Source data 1 raw anti-TUB immunoblot.tif]

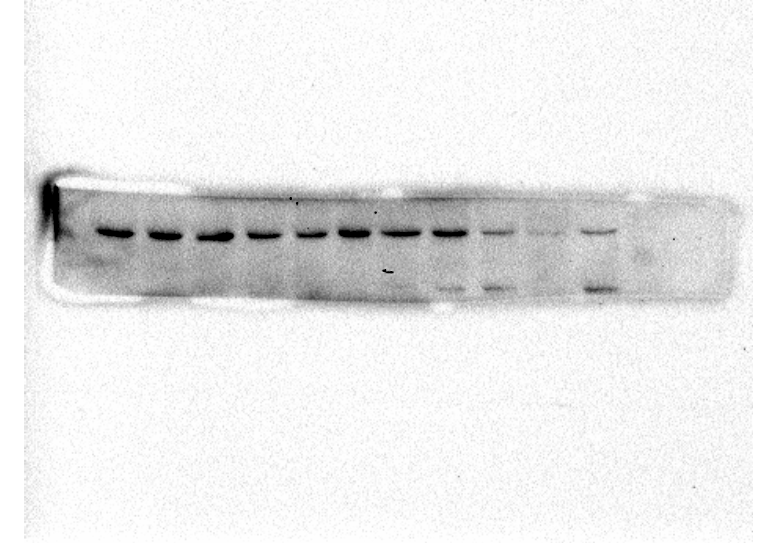

Supplement: Figure 2—source data 3. [file elife-67294-fig2-data3.zip › Figure 2D_Source data 1/Figure 2D-Source data 1 raw anti-CYCB immunoblot.tif]

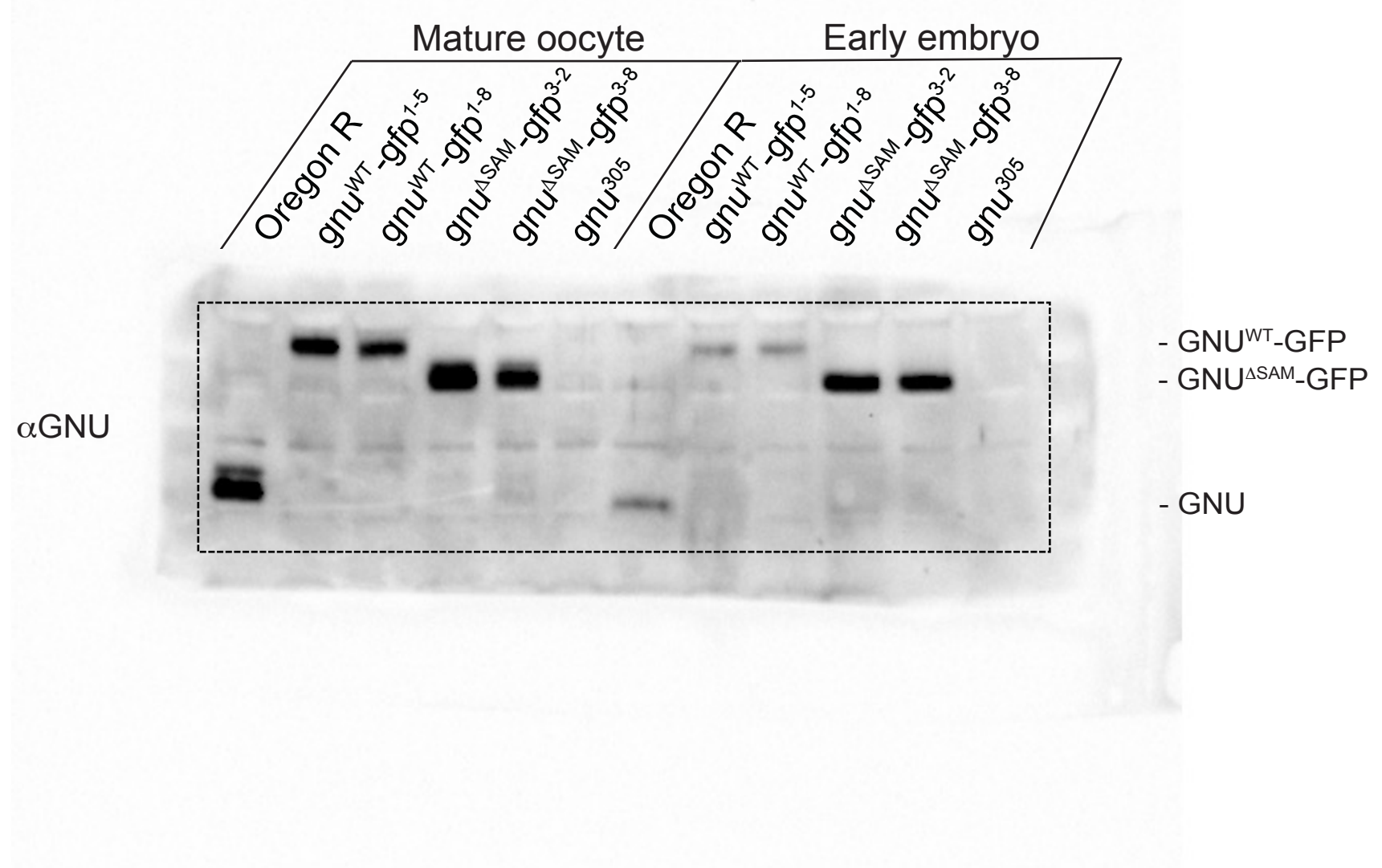

$\alpha$ CYC

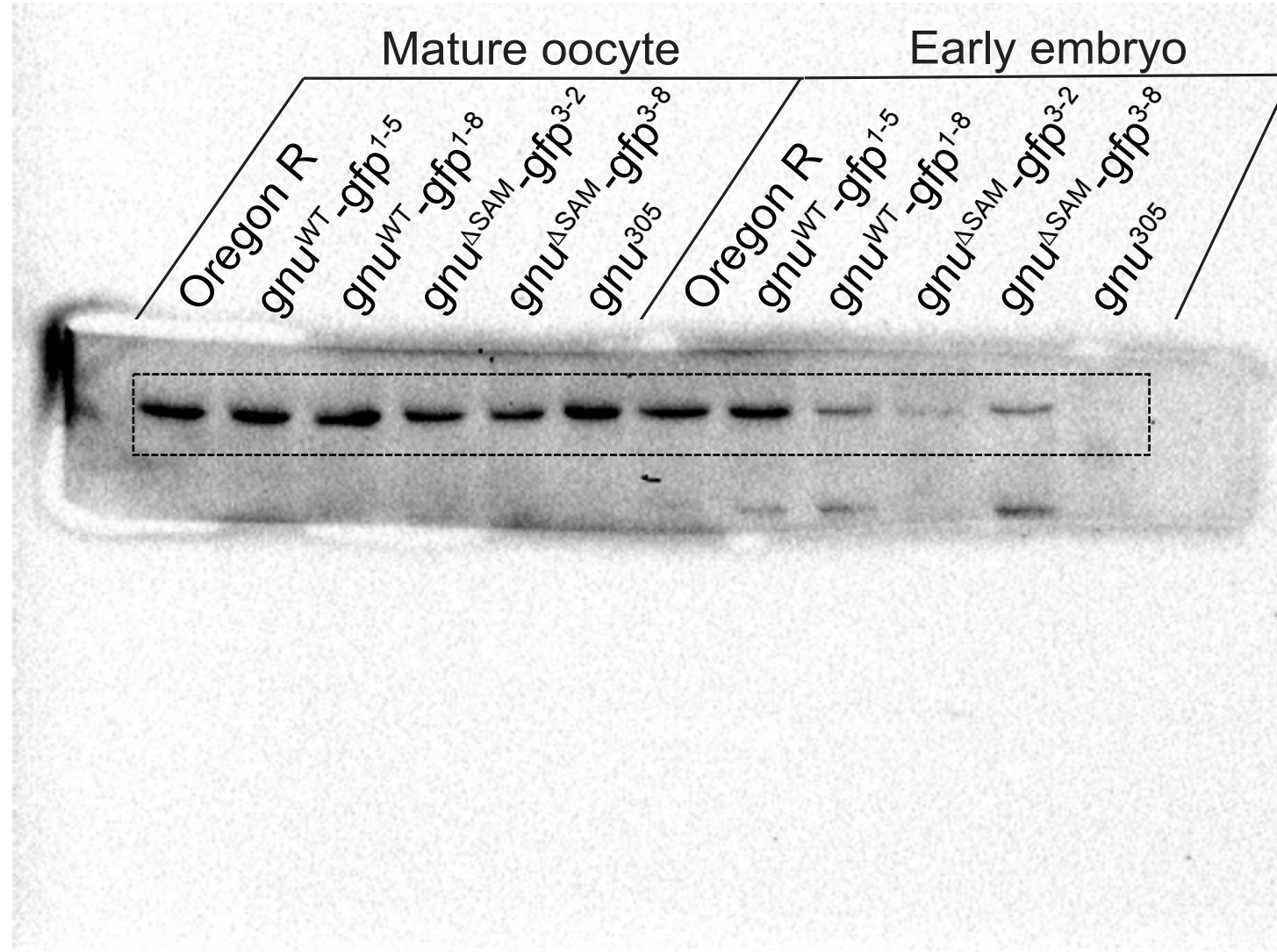

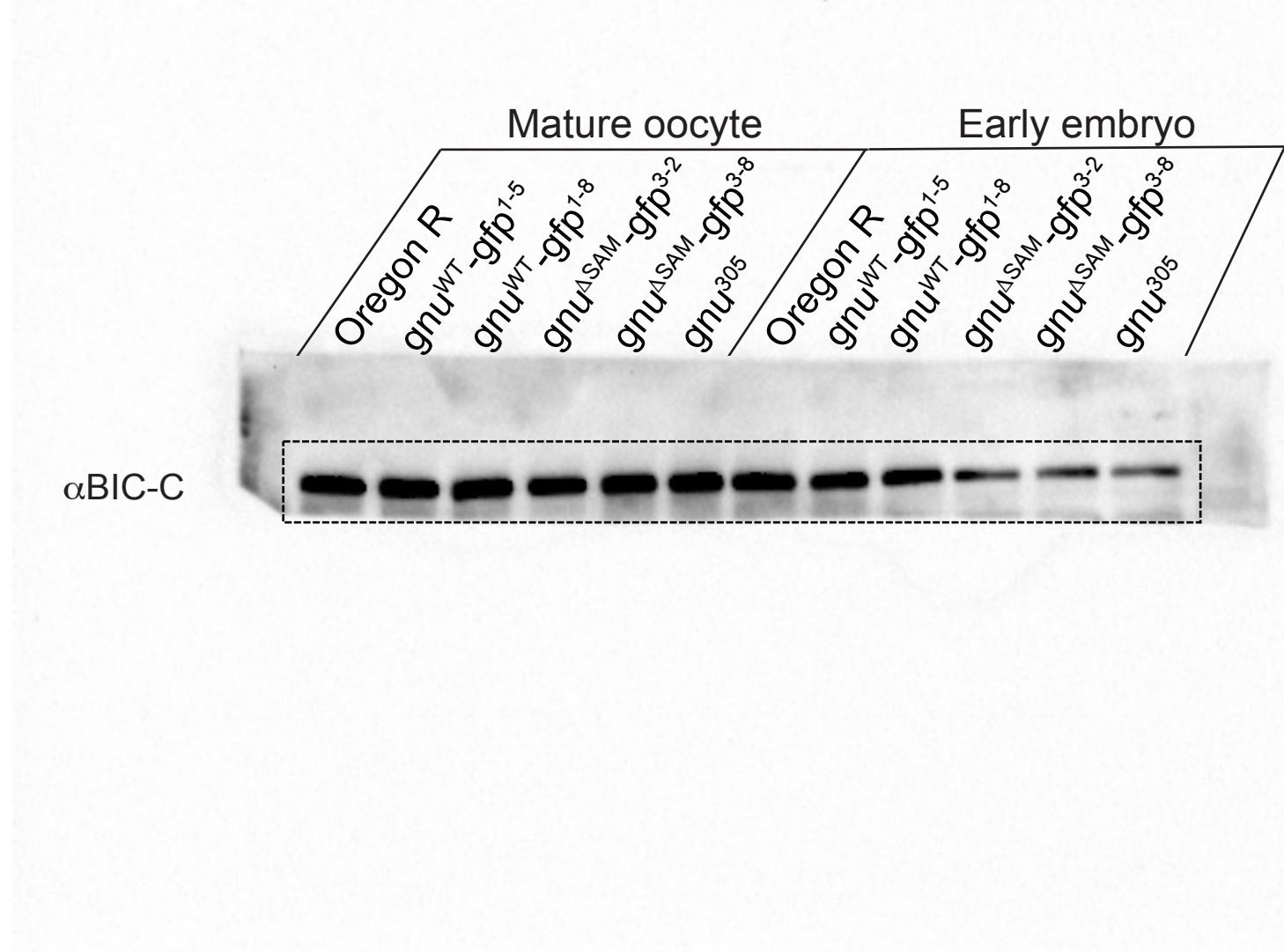

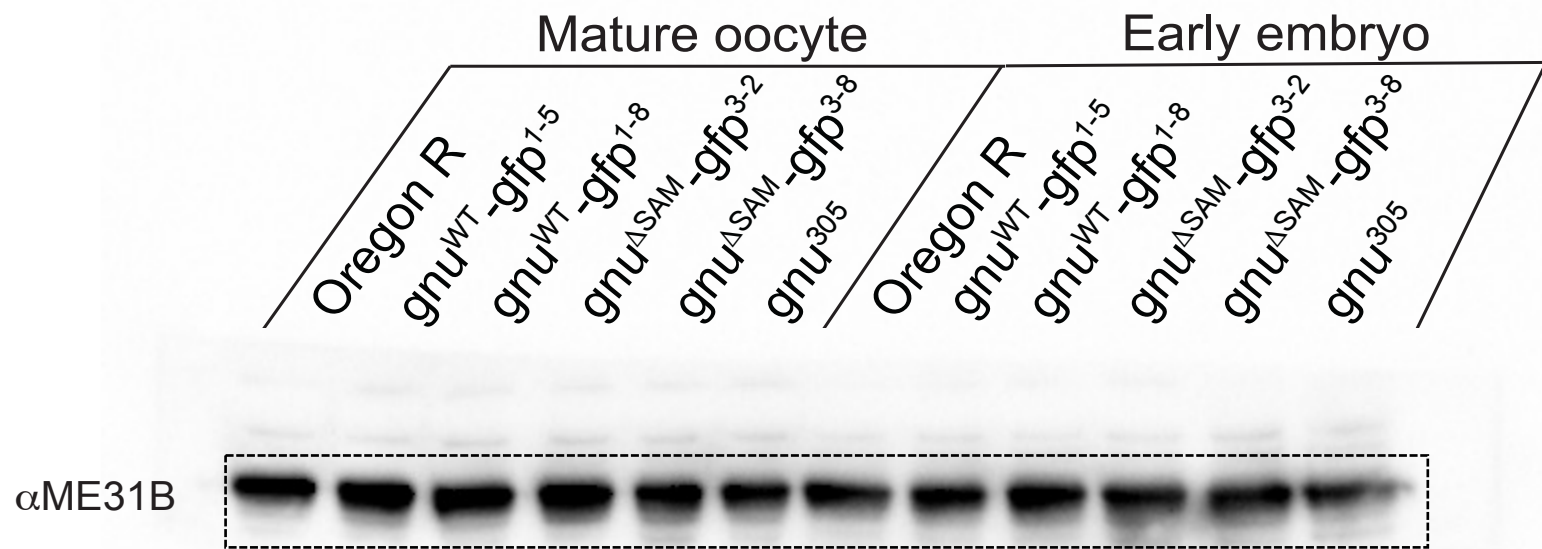

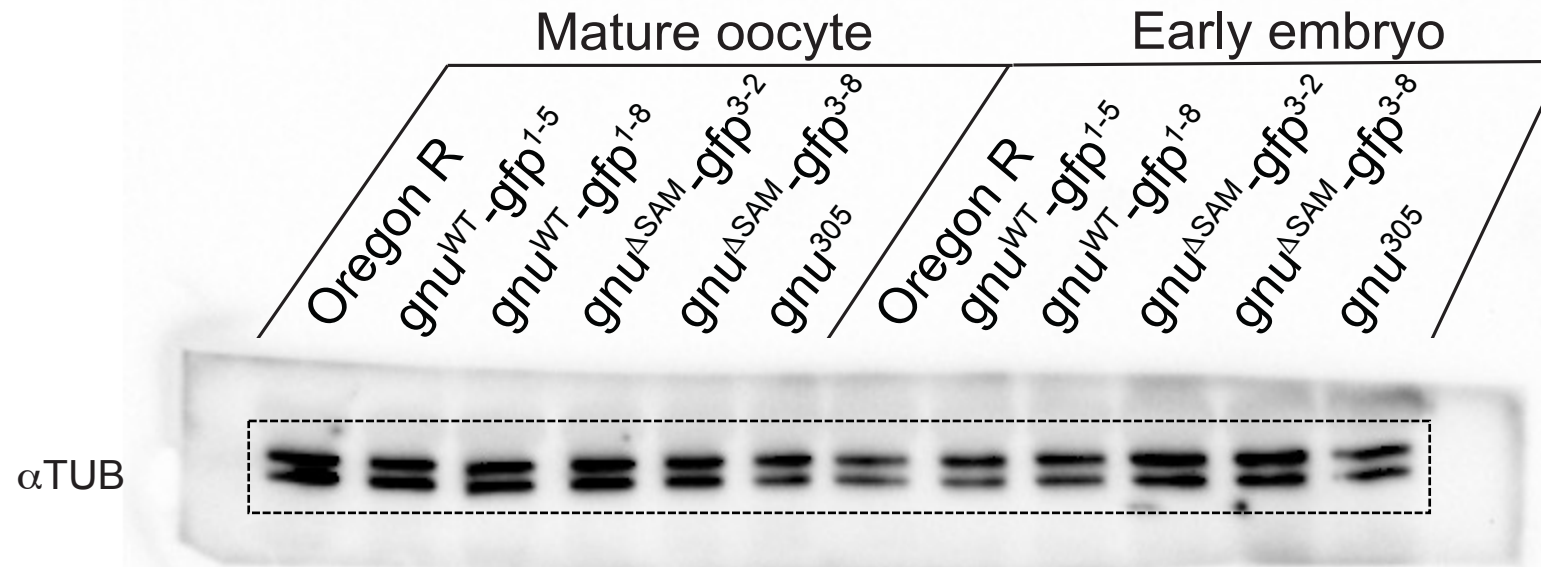

Supplement: Figure 2—source data 3. [file elife-67294-fig2-data3.zip › Figure 2D_Source data 1/Figure 2D-Source data 1 labeled bands.pdf]

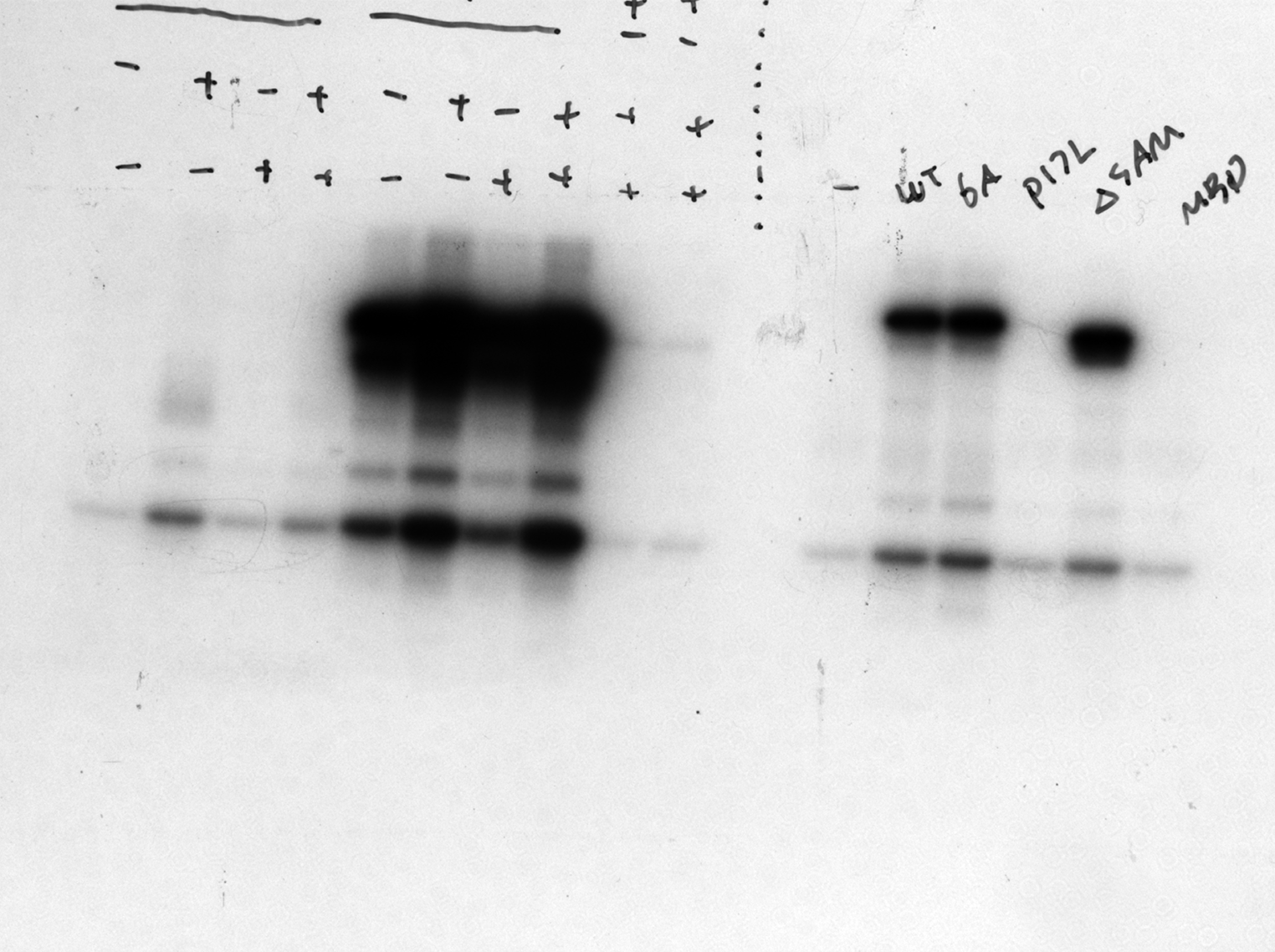

Supplement: Figure 2—source data 4. — Figure with labeled bands. [file elife-67294-fig2-data4.zip › Fig2E_source_data/Figure 2E-Source data 1 Autoradiograph_small.tif]

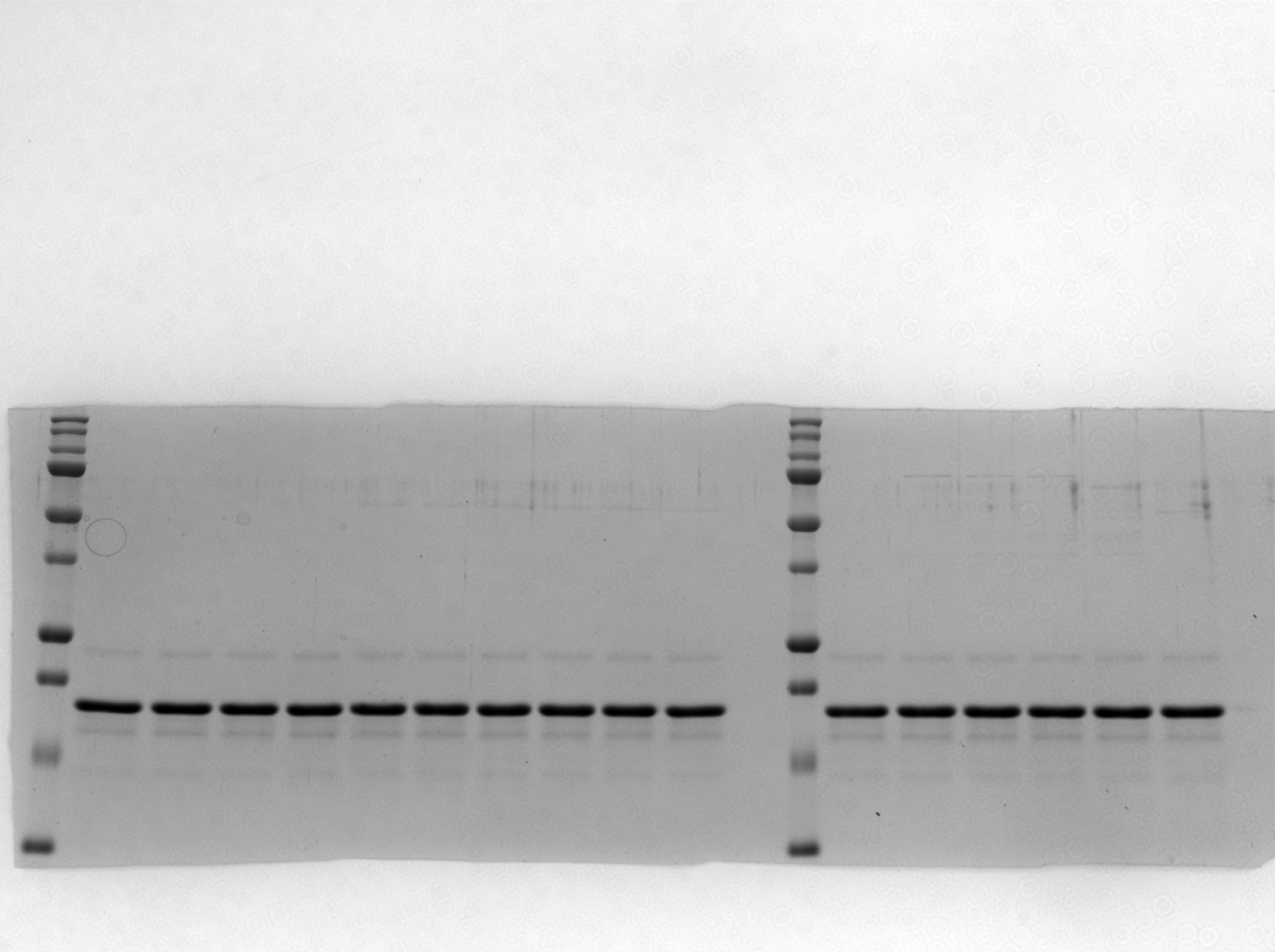

Supplement: Figure 2—source data 4. — Figure with labeled bands. [file elife-67294-fig2-data4.zip › Fig2E_source_data/Figure 2E-Source data 1 coomassie_small.tif]

Figure 2E-Source data

Autoradiograph

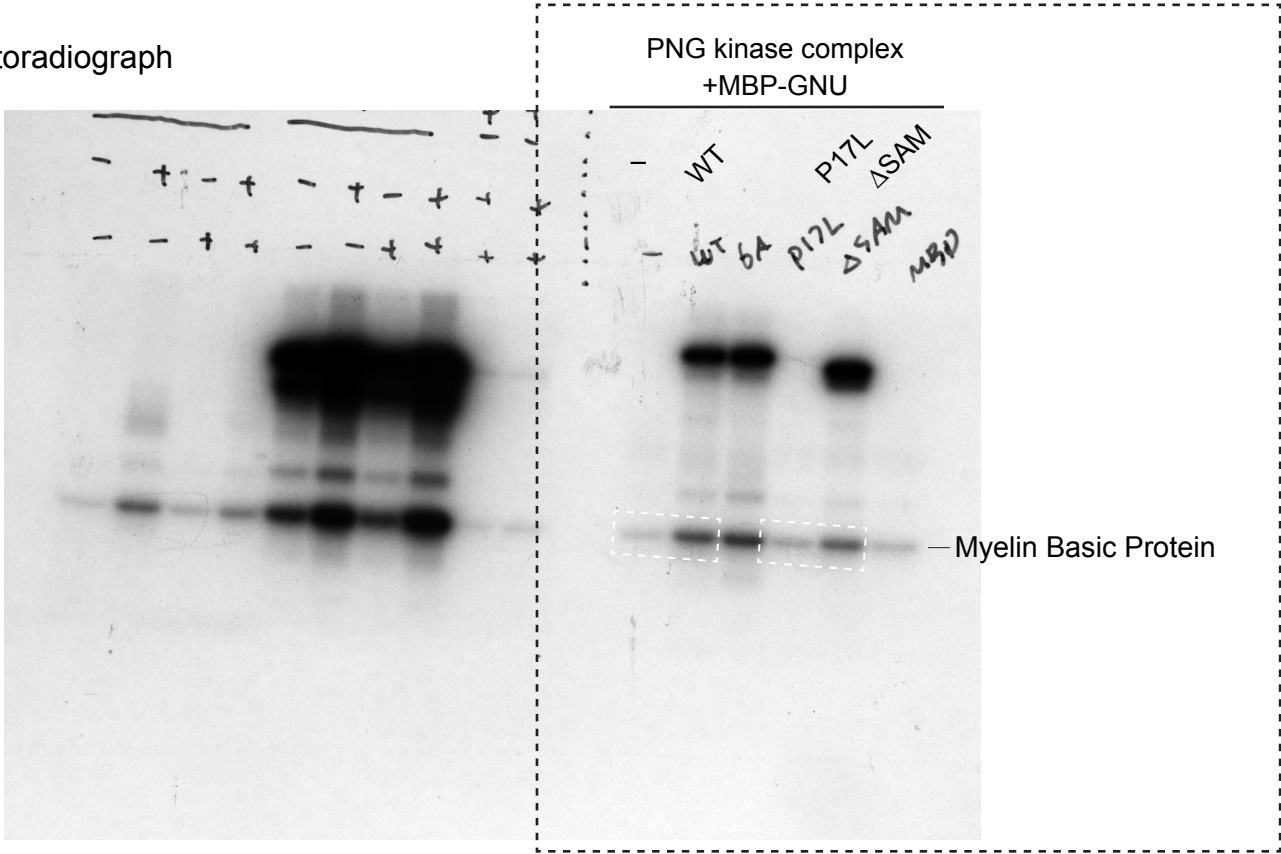

Coomassie

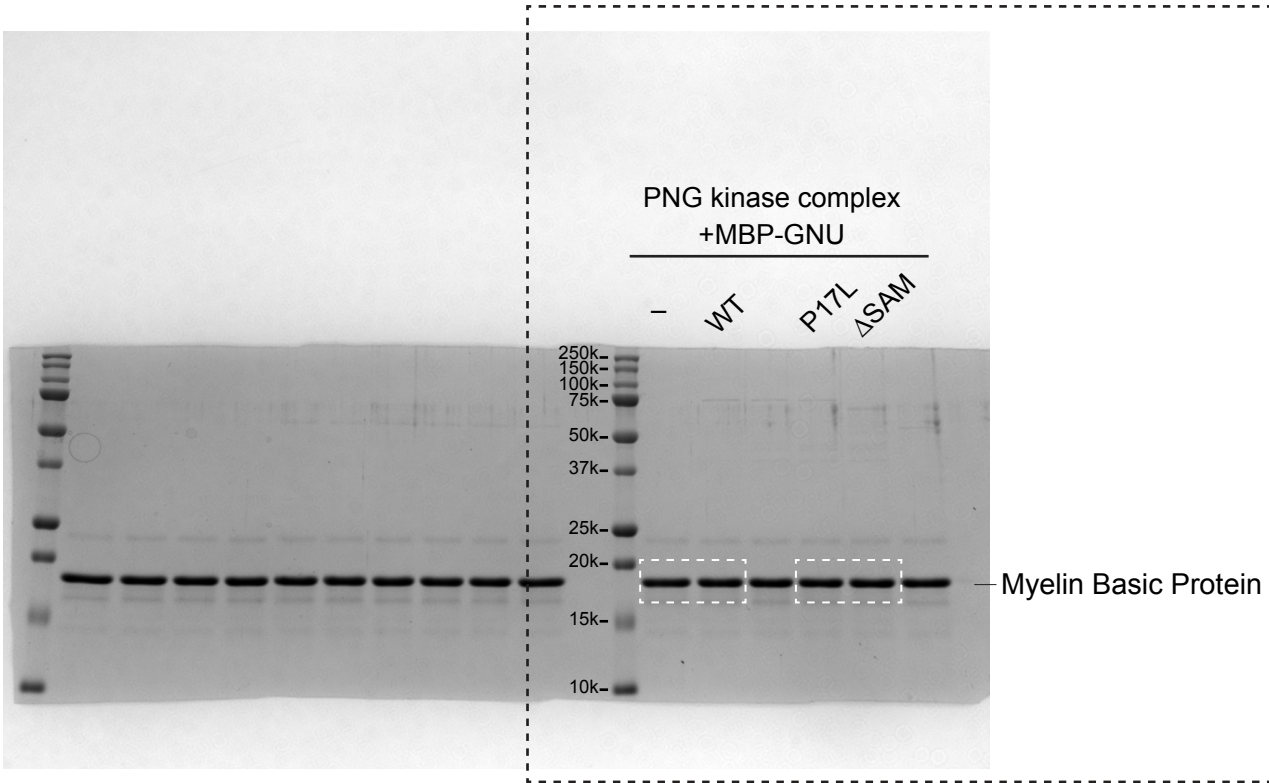

Supplement: Figure 2—source data 4. — Figure with labeled bands. [file elife-67294-fig2-data4.zip › Fig2E_source_data/Figure 2E-Source data 1 labeled bands.pdf]

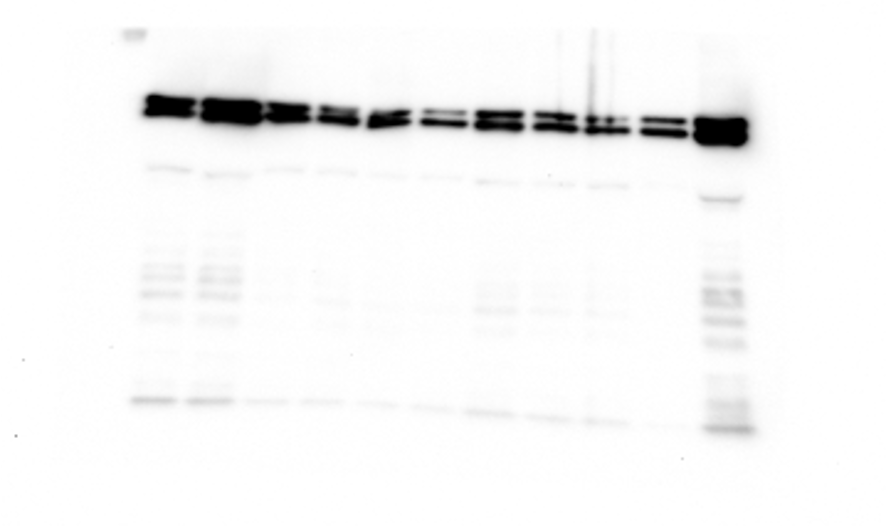

Supplement: Figure 3—figure supplement 1—source data 1. [file elife-67294-fig3-figsupp1-data1.zip › Figure 3_Supplement 1A_Source data 1/Figure 3_Supplement 1A_Source data 1 raw anti-TUB immunoblot.png]

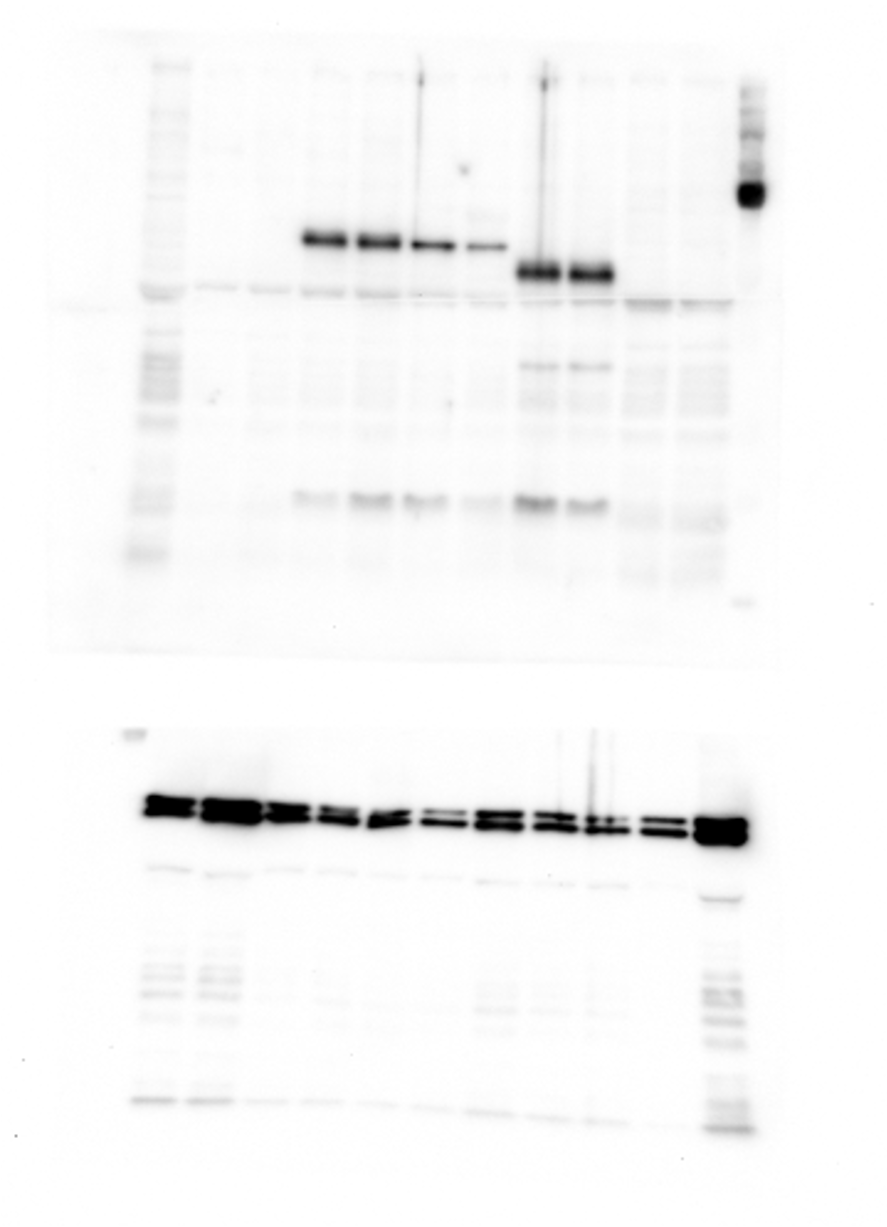

Supplement: Figure 3—figure supplement 1—source data 1. [file elife-67294-fig3-figsupp1-data1.zip › Figure 3_Supplement 1A_Source data 1/Figure 3_Supplement 1A_Source data 1 Original Scan of developed films after exposure.png]

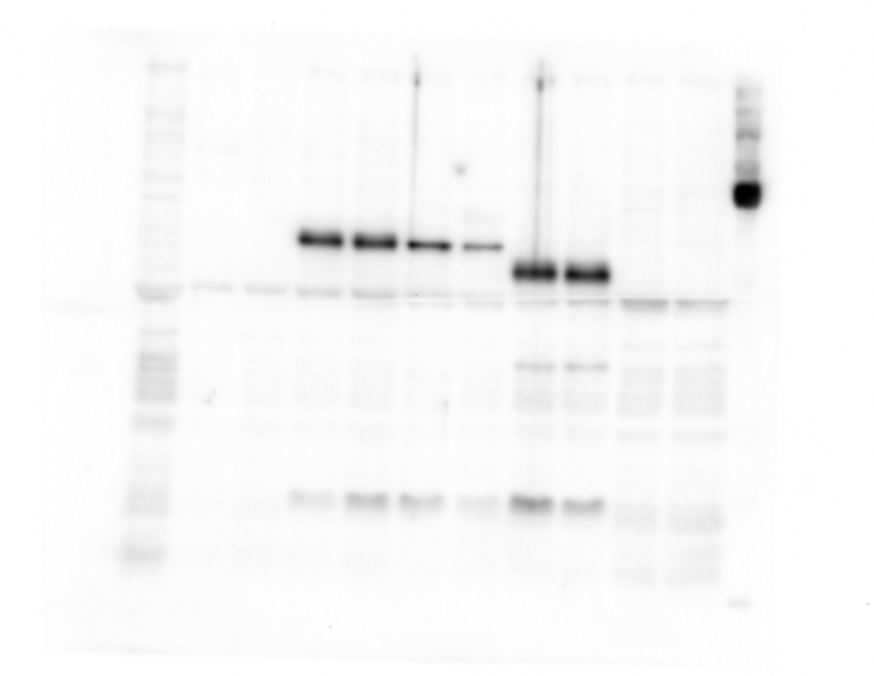

Supplement: Figure 3—figure supplement 1—source data 1. [file elife-67294-fig3-figsupp1-data1.zip › Figure 3_Supplement 1A_Source data 1/Figure 3_Supplement 1A_Source data 1 raw anti-GFP immunoblot.png]

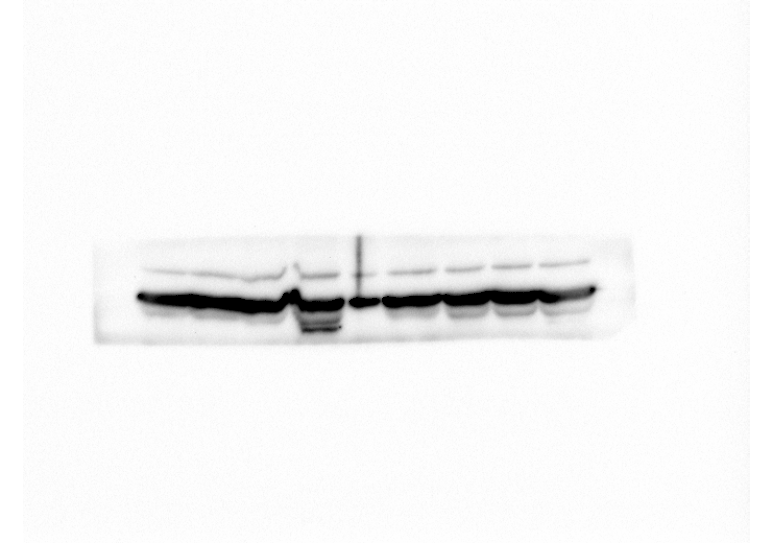

Supplement: Figure 5—figure supplement 1—source data 1. [file elife-67294-fig5-figsupp1-data1.zip › Figure 5_Supplement 1A_Source data 1/Figure 5-Supplement 1A-Source data 1 raw anti-ME31B immunoblot.tif]

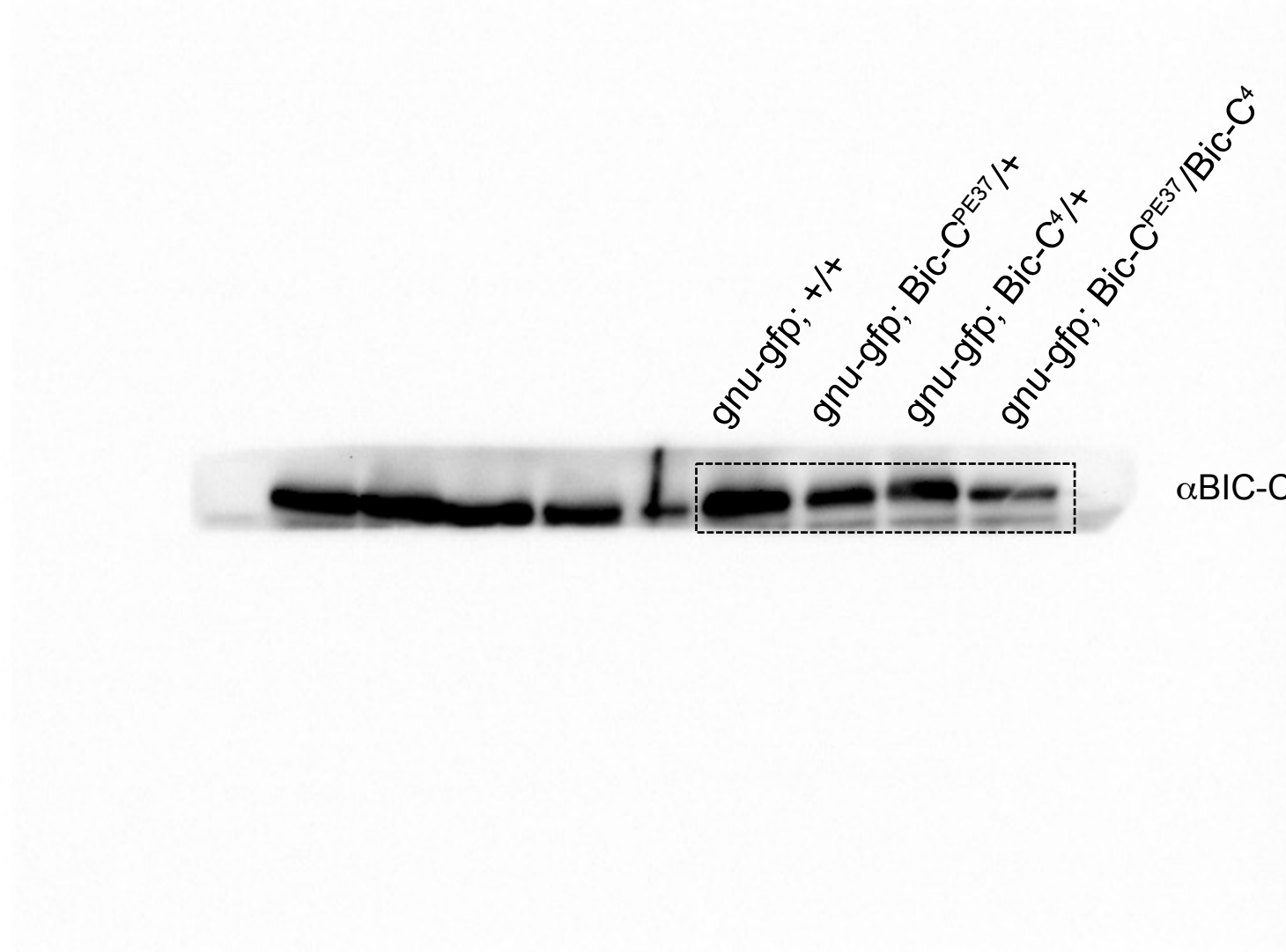

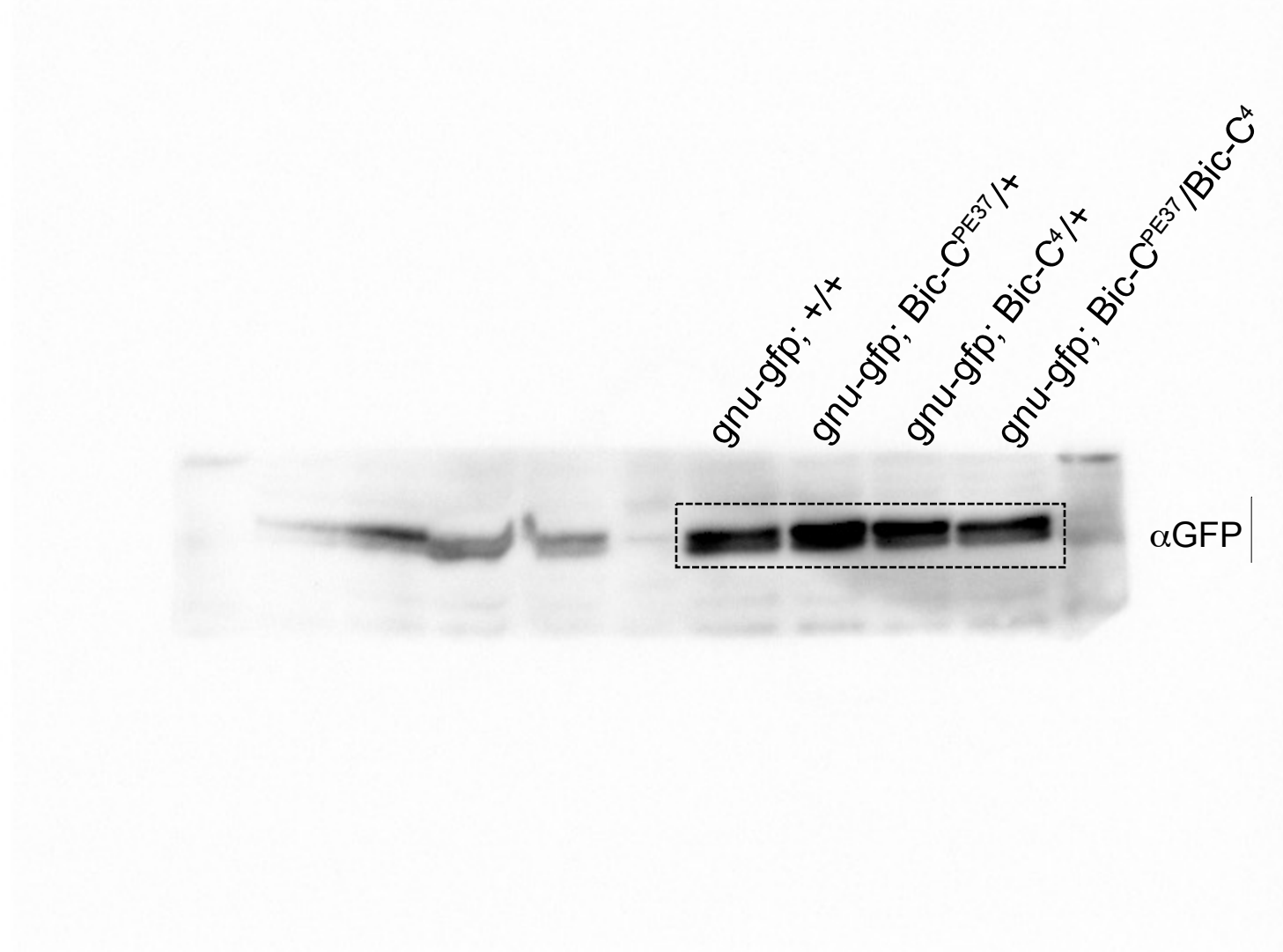

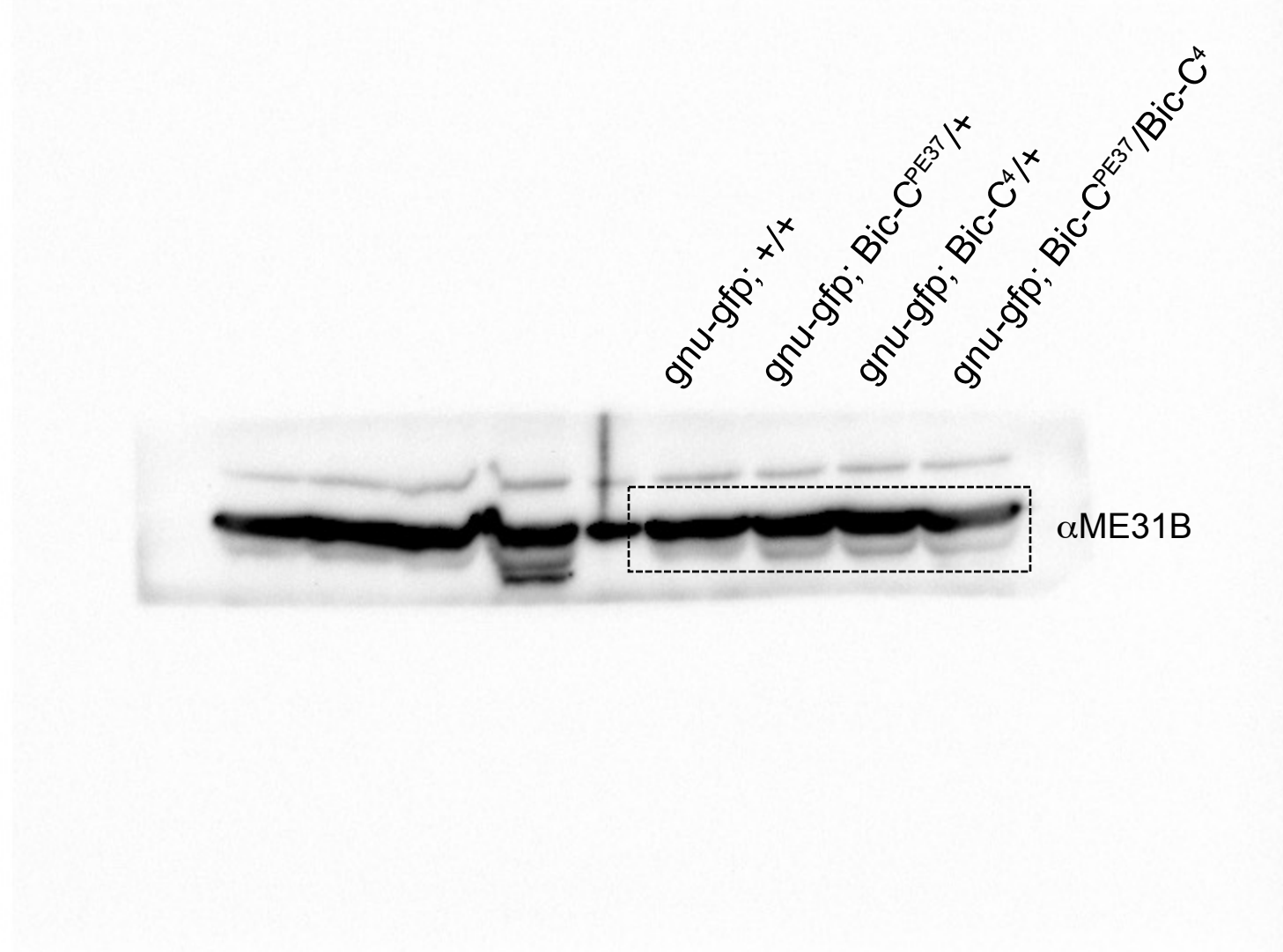

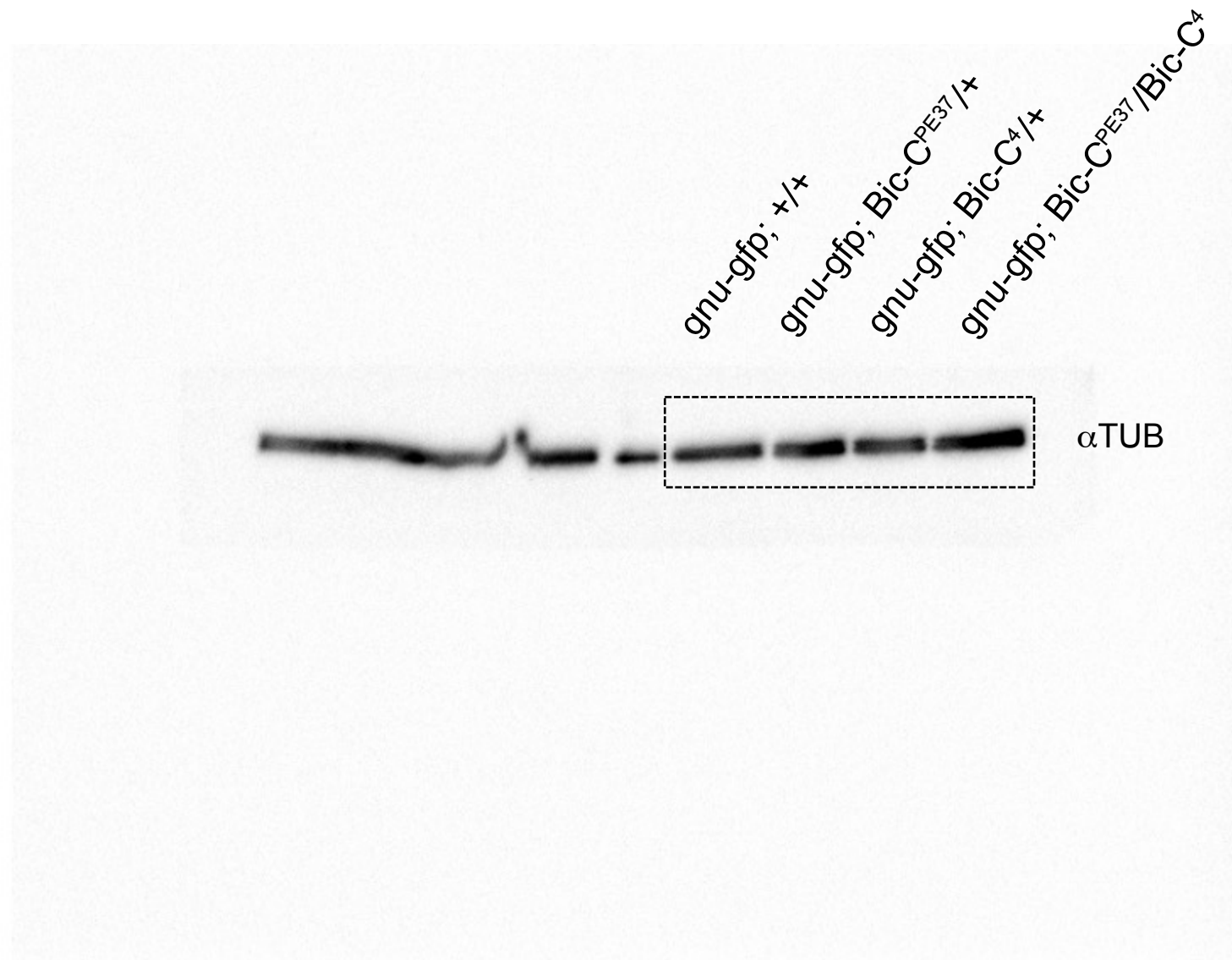

Supplement: Figure 5—figure supplement 1—source data 1. [file elife-67294-fig5-figsupp1-data1.zip › Figure 5_Supplement 1A_Source data 1/Figure 5-Supplement 1A-Source data 1 labeled bands.pdf]

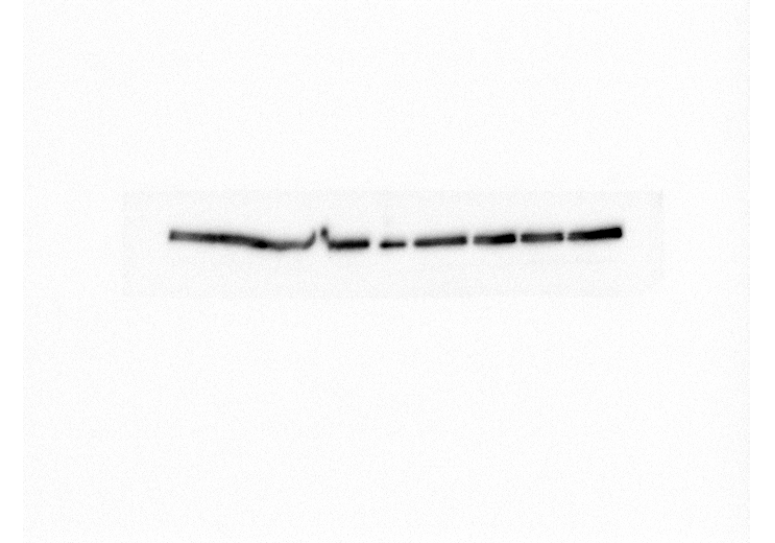

Supplement: Figure 5—figure supplement 1—source data 1. [file elife-67294-fig5-figsupp1-data1.zip › Figure 5_Supplement 1A_Source data 1/Figure 5-Supplement 1A-Source data 1 raw anti-TUB immunoblot.tif]

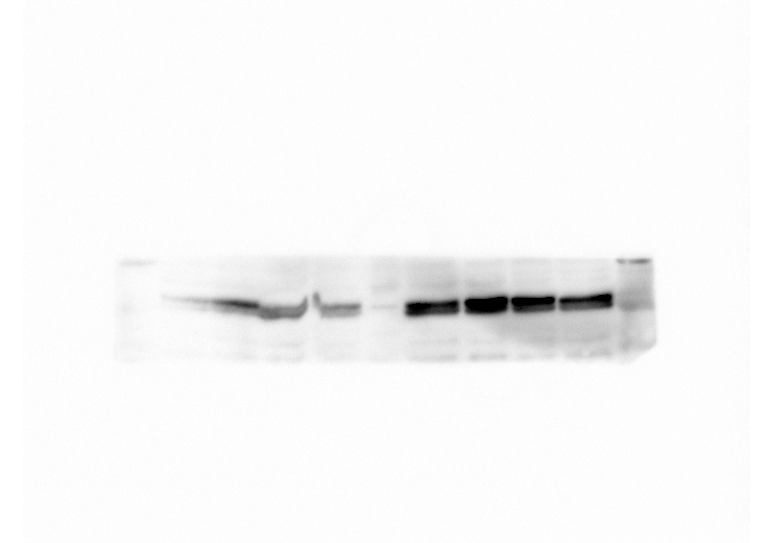

Supplement: Figure 5—figure supplement 1—source data 1. [file elife-67294-fig5-figsupp1-data1.zip › Figure 5_Supplement 1A_Source data 1/Figure 5-Supplement 1A-Source data 1 raw anti-GFP immunoblot.tif]

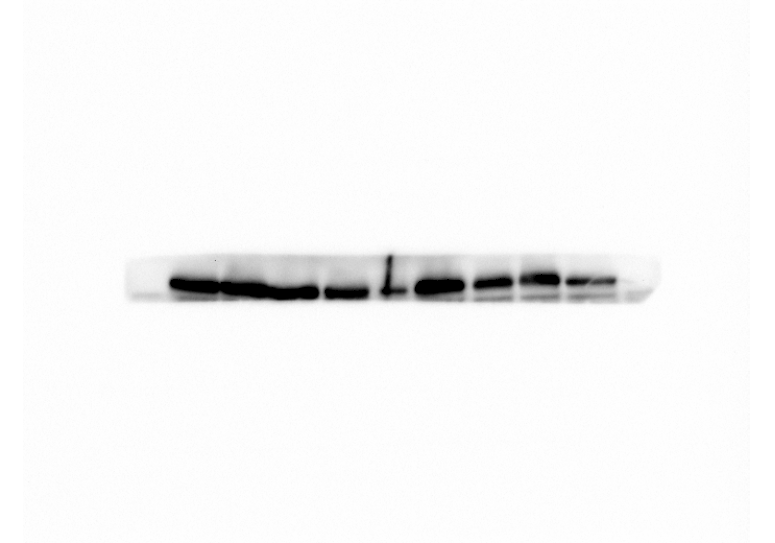

Supplement: Figure 5—figure supplement 1—source data 1. [file elife-67294-fig5-figsupp1-data1.zip › Figure 5_Supplement 1A_Source data 1/Figure 5-Supplement 1A-Source data 1 raw anti-BIC-C immunoblot.tif]

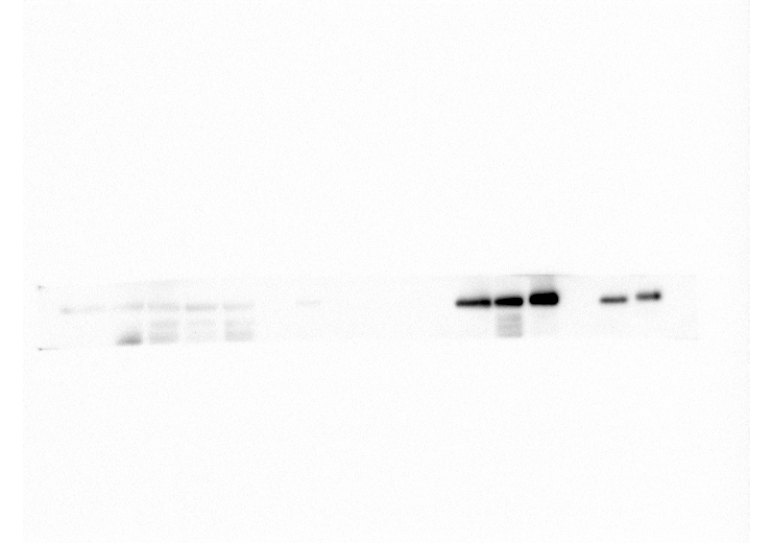

Supplement: Figure 6—source data 2. [file elife-67294-fig6-data2.zip › Figure 6A_Source data 2/Figure 6A-Source data 1 raw anti-BIC-C immunoblot.tif]

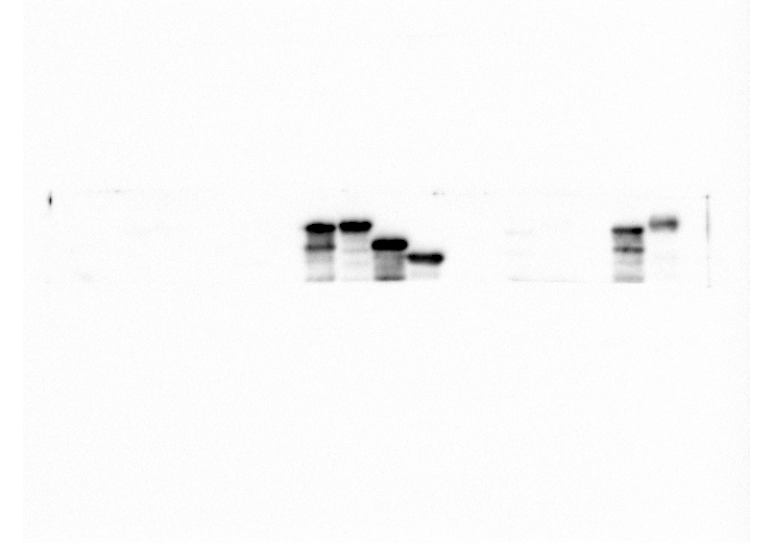

Supplement: Figure 6—source data 2. [file elife-67294-fig6-data2.zip › Figure 6A_Source data 2/Figure 6A-Source data 1 raw anti-GFP immunoblot.tif]

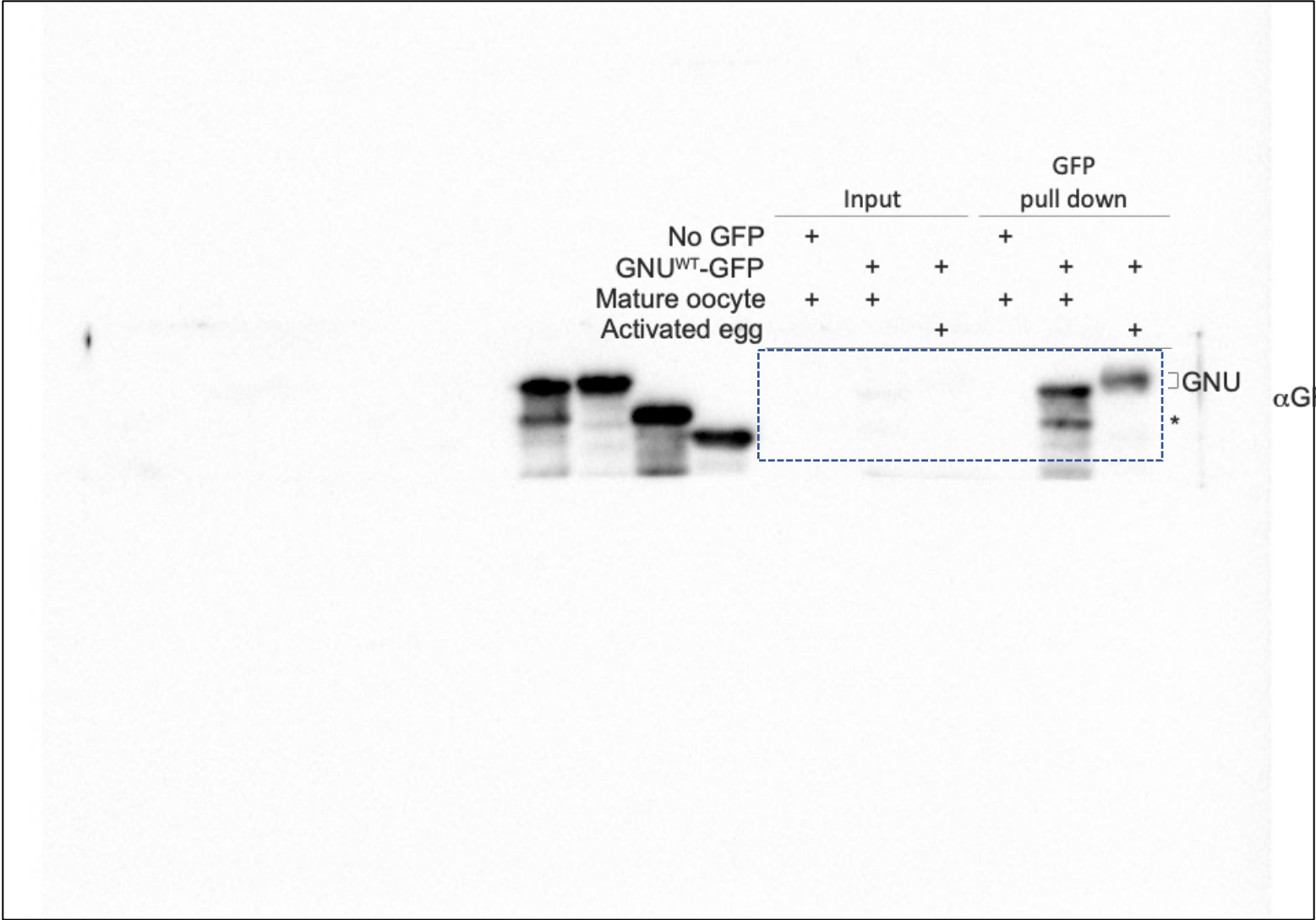

|                        | Input |   |   | GFP<br>pull down |   |   |
|------------------------|-------|---|---|------------------|---|---|
| No GFP                 | +     |   |   | +                |   |   |
| GNU <sup>WT</sup> -GFP |       | + | + |                  | + | + |
| Mature oocyte          | +     | + |   | +                | + |   |
| Activated egg          |       |   | + |                  |   | + |

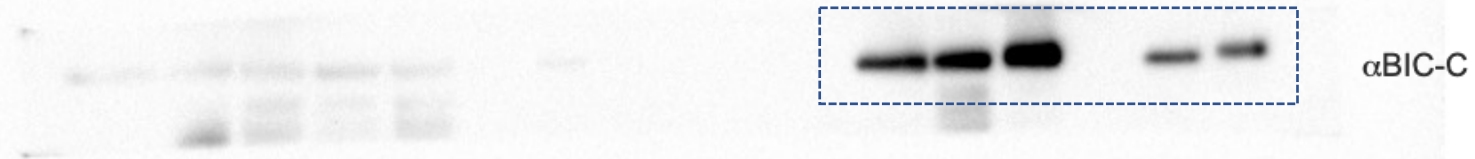

Supplement: Figure 6—source data 2. [file elife-67294-fig6-data2.zip › Figure 6A_Source data 2/Figure 6A-Source data 1 labeled bands.pdf]
