# Supplementary material for: The GNU subunit of PNG kinase, the developmental regulator of mRNA translation, binds BIC-C to localize to RNP granules
Source: eLife. 2021 Jul 12;10:e67294. doi: 10.7554/eLife.67294 (PMC8313231; doi:10.7554/eLife.67294)
Supplement: Figure 3—figure supplement 1—source data 1. [file elife-67294-fig3-figsupp1-data1.zip › Figure 3_Supplement 1A_Source data 1/Figure 3_Supplement 1A_Source data 1 labeled bands.pdf]

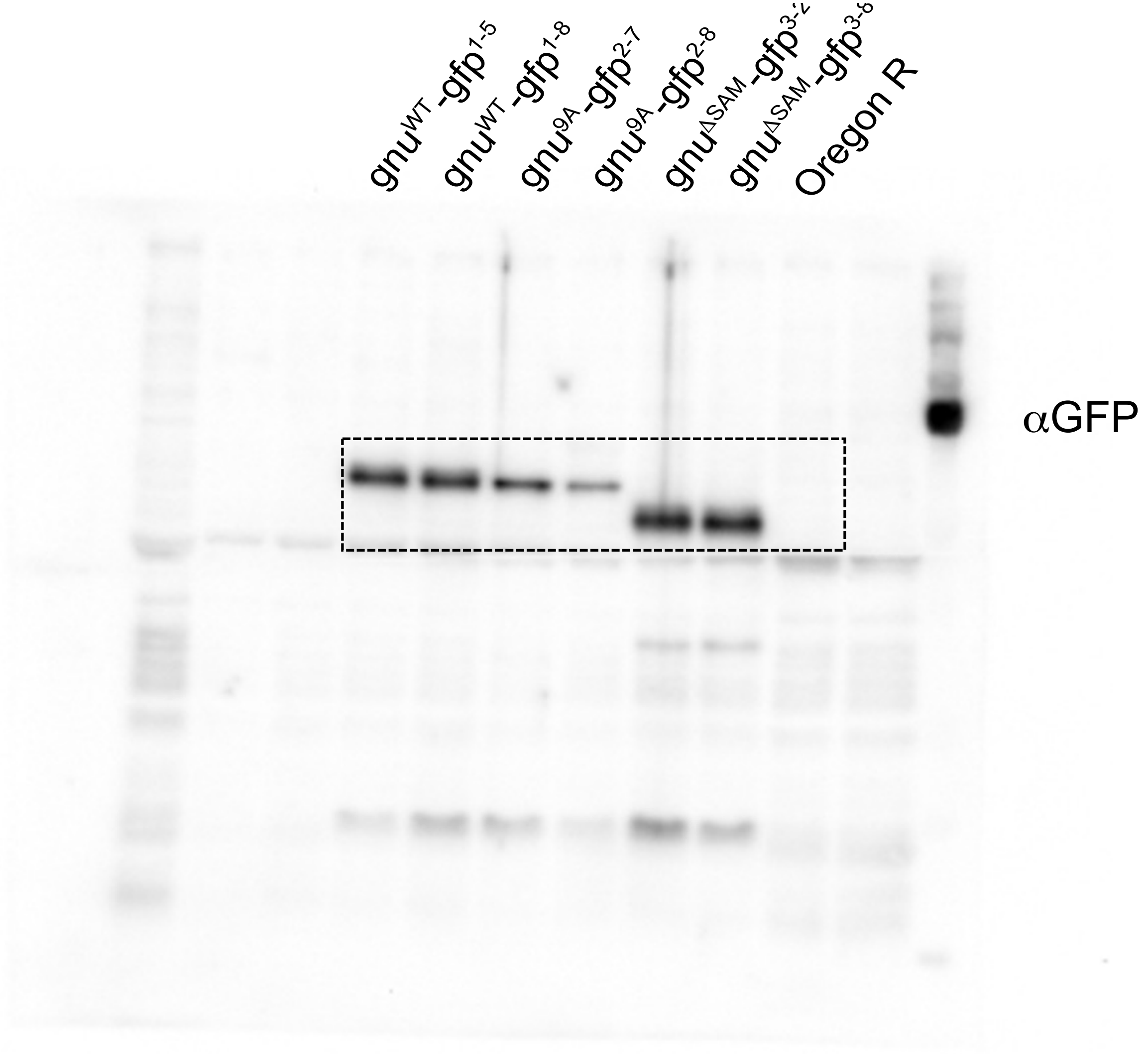
gnu^{WT}-gfp^{1-5}
gnu^{WT}-gfp^{1-8}
gnu^{9A}-gfp^{2-7}
gnu^{9A}-gfp^{2-8}
$gnu^{\Delta SAM}$-gfp^{3-2}
$gnu^{\Delta SAM}$-gfp^{3-8}
Oregon R
αGFP

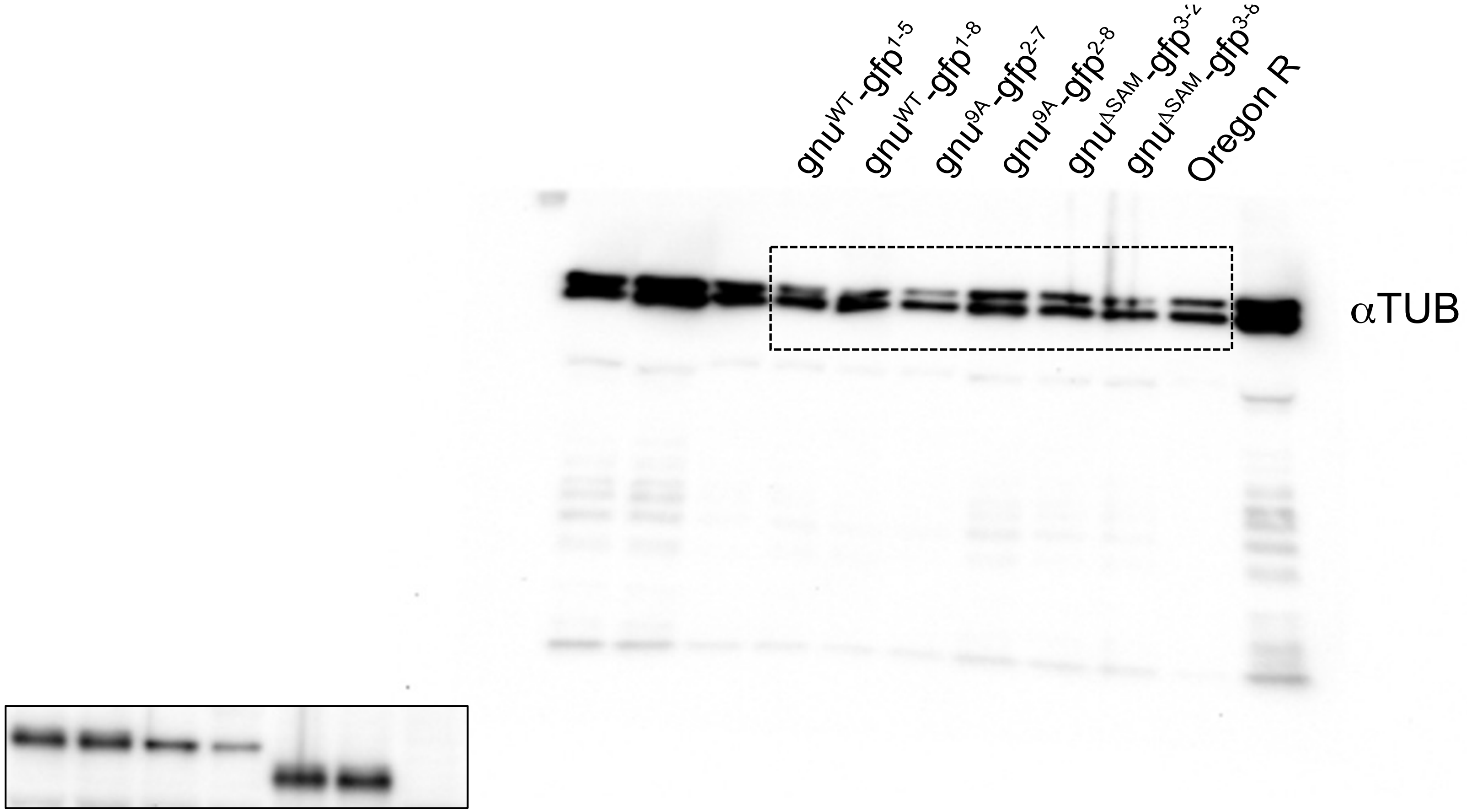
gnuWT-gfp1-5
gnuWT-gfp1-8
gnu9A-gfp2-7
gnu9A-gfp2-8
gnuΔSAM-gfp3-2
gnuΔSAM-gfp3-8
Oregon R
αTUB
