# Supplementary material for: The GNU subunit of PNG kinase, the developmental regulator of mRNA translation, binds BIC-C to localize to RNP granules
Source: eLife. 2021 Jul 12;10:e67294. doi: 10.7554/eLife.67294 (PMC8313231; doi:10.7554/eLife.67294)
Supplement: Supplementary file 1. — The fold enrichment in GNU-GFP over a no GFP control is shown. The source data for this table are in Figure 1—figure supplement 1—source data 1. [file elife-67294-supp1.docx]

| **Supplementary Table 1. Interactors with GNU in mature oocytes identified by IP-MS** | | |
| --- | --- | --- |
| **Protein** | **Rank** | **Fold enrichment in GNU-GFP**  **over no GFP control** |
| **BIC-C** | 1 | 23.17 |
| **TWS** | 2 | 22.30 |
| **YPS** | 3 | 21.58 |
| **RpS6** | 4 | 21.13 |
| **TYF** | 5 | 21.09 |
| **ATX-2** | 6 | 21.07 |
| **PP2A-29B** | 7 | 20.96 |
| **RpL21** | 8 | 20.72 |
| **IGF-II mRNA-binding Protein (IMP)** | 9 | 20.67 |
| **CG15735** | 10 | 20.52 |
| **CG5726** | 11 | 20.34 |
| **MTS** | 12 | 20.33 |
| **BOR** | 13 | 20.33 |
| **Tcp1-like** | 14 | 20.14 |
| **RDX** | 15 | 19.98 |
| **GAPcenA** | 16 | 19.97 |
| **SCPX** | 17 | 19.89 |
| **FMR1** | 18 | 19.88 |
| **Aspartyl-tRNA synthetase** | 19 | 19.85 |
| **CG4586** | 20 | 19.65 |
| **NCD** | 21 | 19.56 |
| **PEX14** | 22 | 19.48 |
| **T-cp1ζ** | 23 | 19.34 |
| **Glutamyl-prolyl-tRNA Synthetase** | 24 | 19.24 |
| **RpS24** | 25 | 19.22 |
| **RpL17** | 26 | 19.22 |
| **CG17018** | 27 | 19.18 |
| **LON** | 28 | 19.11 |
| **LARP** | 29 | 18.95 |
| **SAS-4** | 30 | 18.92 |
| **Isoleucyl-tRNA Synthetase** | 31 | 18.77 |
| **SWA** | 32 | 18.69 |
| **MEGATOR** | 33 | 18.49 |
| **γTUB37C** | 34 | 18.37 |
| **LOST** | 35 | 18.3 |
| **Lysyl-tRNA Synthetase** | 36 | 18.27 |
| **WISP** | 37 | 18.23 |
| **TACC** | 38 | 17.79 |
| **GRIP71** | 39 | 17.62 |
| **CP18** | 40 | 17.55 |
| **BON** | 41 | 17.52 |
| **AGO2** | 42 | 17.5 |
| **ZIP** | 43 | 17.26 |
| **ADP ribosylation factor at 102F** | 44 | 16.6 |
| **GNU** | 45 | 7.16 |

Source data for this table are in Figure 1-source data 2.
